# Hydrogel-Based Fiber Biofabrication Techniques for Skeletal Muscle Tissue Engineering

**DOI:** 10.1021/acsbiomaterials.1c01145

**Published:** 2022-01-27

**Authors:** Marina Volpi, Alessia Paradiso, Marco Costantini, Wojciech Świȩszkowski

**Affiliations:** † Faculty of Materials Science and Engineering, Warsaw University of Technology, Warsaw 02-507, Poland; ‡ Institute of Physical Chemistry, Polish Academy of Sciences, Warsaw 01-224, Poland

**Keywords:** skeletal muscle tissue engineering, hydrogels, cell alignment, electrospinning, 3D bioprinting, microfluidic spinning

## Abstract

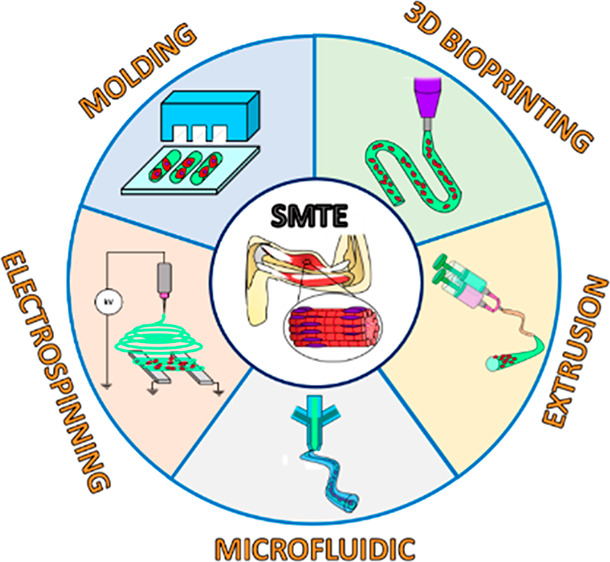

The functional capabilities of skeletal muscle are strongly correlated with its well-arranged microstructure, consisting of parallelly aligned myotubes. In case of extensive muscle loss, the endogenous regenerative capacity is hindered by scar tissue formation, which compromises the native muscle structure, ultimately leading to severe functional impairment. To address such an issue, skeletal muscle tissue engineering (SMTE) attempts to fabricate *in vitro* bioartificial muscle tissue constructs to assist and accelerate the regeneration process. Due to its dynamic nature, SMTE strategies must employ suitable biomaterials (combined with muscle progenitors) and proper 3D architectures. In light of this, 3D fiber-based strategies are gaining increasing interest for the generation of hydrogel microfibers as advanced skeletal muscle constructs. Indeed, hydrogels possess exceptional biomimetic properties, while the fiber-shaped morphology allows for the creation of geometrical cues to guarantee proper myoblast alignment. In this review, we summarize commonly used hydrogels in SMTE and their main properties, and we discuss the first efforts to engineer hydrogels to guide myoblast anisotropic orientation. Then, we focus on presenting the main hydrogel fiber-based techniques for SMTE, including molding, electrospinning, 3D bioprinting, extrusion, and microfluidic spinning. Furthermore, we describe the effect of external stimulation (i.e., mechanical and electrical) on such constructs and the application of hydrogel fiber-based methods on recapitulating complex skeletal muscle tissue interfaces. Finally, we discuss the future developments in the application of hydrogel microfibers for SMTE.

## 1 Introduction

Skeletal muscle comprises approximately 45% of the adult human body weight, representing the largest tissue type in the body. Skeletal muscle is mainly responsible for generating contractile forces, ensuring the functional performance of many critical physiological functions, including locomotion, mastication, and ocular movements.^1,2^ The functional capabilities of skeletal muscle tissue are strongly connected with its well-arranged microstructure, which consists of uniaxially oriented and densely packed myofibers.^3,4^ Myofibers initially originate during myogenesis from the progressive fusion of undifferentiated myogenic precursor cells (i.e., myoblasts) into elongated and multinucleated myotubes, which subsequently mature into muscle fibers composed of many parallel myofibrils.^5^ The basic unit of a myofibril is the sarcomere, composed of highly organized intracellular myofilaments (e.g., actin filaments, myosin filaments) sliding on each other to generate muscle contraction and relaxation.^6^ In general, skeletal muscle possesses an endogenous capacity to regenerate in response to minor damages resulting from muscle tears, small lacerations, strains, or toxins.^7^ The regeneration process involves the activation of resident multipotent stem cells (i.e., myosatellite cells), which undergo proliferation and subsequently differentiate into myoblasts, ultimately fusing to form myofibers and integrate into the unimpaired muscle tissue.^8^ However, skeletal muscle cannot restore extensive damages (i.e., muscle defects larger than 20% of the original mass), resulting from traumatic injuries, aggressive malignant tumor excisions, muscle denervation, or skeletal muscle degenerative diseases.^9^ In this case, the innate regeneration process is hindered by the formation of fibrous scar tissue, which in turn results in a loss of the native biological composition and microstructure, thus leading to severe functional impairment.^8^ Among the treatment options available for skeletal muscle restoration, the current clinical standard consists of the engraftment of healthy autologous tissues (i.e., muscle flap). However, this approach includes several limitations such as a shortage of donor tissue, loss of function at the donor site, and donor-site morbidity.^10^ In this scenario, SMTE provides a more promising alternative to the current clinical standard treatment. Indeed, SMTE strategies offer the possibility to produce *in vitro* bioartificial constructs, which can potentially support and accelerate the regeneration process.^11^ To successfully engineer a skeletal muscle tissue construct, SMTE aims to combine a suitable biomaterial substrate and a proper scaffold design.^12−14^ As a biomaterial scaffold, synthetic polymers (e.g., polycaprolactone (PCL), polylactic acid (PLA), poly(lactic-*co*-glycolic acid) (PLGA)) have been proven to efficiently support skeletal muscle tissue regeneration.^8,15^ However, the cytotoxicity issues mostly related to residual solvents employed during scaffold manufacturing and the lack of cell-supportive nature have prompted alternative biomaterials with higher biocompatibility and biomimetic properties.^16^ Among those, hydrogel-based biomaterials are considered promising candidates for engineering skeletal muscle tissue.^17^ Indeed, hydrogels possess unique biomimetic properties, including higher water content, biochemical cues, and tunable physical and mechanical properties.^18^ Moreover, hydrogels also possess the amenability to be easily processed in different design configurations by applying various advanced microfabrication techniques.^19−23^ In this frame, fiber-based tissue engineering techniques enable the fabrication of hydrogel microfibers, representing an optimal platform to simultaneously provide a highly cell-compatible microenvironment and a structured directionality toward proper muscle tissue development.^24,25^ Moreover, hydrogel microfibers can be used both as a single self-standing structure and as a building block unit that can be further assembled to rebuild full-scale 3D skeletal muscle tissue constructs.^26^ In this review, we present a critical overview of the current state-of-the-art of hydrogel-based fiber biofabrication techniques for engineering skeletal muscle tissue. First, the most promising hydrogels and conventional engineering methods for *in vitro* myoblast alignment are introduced. Then, the focus is shifted to the main advances in the fabrication methods of hydrogel-based microfibers for SMTE. Furthermore, the effect of mechanical and electrical stimulation on fibrous hydrogel-based constructs is described. Finally, hydrogel fiber fabrication methods for engineering complex skeletal muscle tissue interfaces are also presented.

## 2 Hydrogels for SMTE

Hydrogels constitute a class of polymeric materials characterized by a hydrophilic structure that allows the storage of a large amount of water in a three-dimensional network. Hydrogels own unique biomimetic properties, including high permeability, biocompatibility, and tunable mechanical properties. Moreover, they can be easily functionalized to closely recapitulate the intrinsic features of a specific biological tissue.^20^ For such attractive characteristics, hydrogels represent the first-choice biomaterials for SMTE.^17^ In this section, we provide an overview of the hydrogels most commonly used in SMTE and their cross-linking methods (Table 1). Moreover, we present the main hydrogel properties including biochemical, mechanical, and electroconductive ones, which enable recapitulation of a suitable biomimetic microenvironment for skeletal muscle tissue development.

**Table 1:** Summary of Common Hydrogels, Their Cross-Linking Methods, and Main Properties Used for SMTE

hydrogel	natural/synthetic	thermal gelation	cross-linking agents	main properties	ref
collagen	natural	*T* = 37 °C	genipin, microbial transglutaminase	cell adhesives sites	
				highly biodegradability	(32, 33, 36)
gelatin	natural	*T* < 30–35 °C	genipin, microbial transglutaminase	cell adhesive sites	(34−36)
				low immunogenicity compared to collagen	
				highly biodegradability	
fibrinogen	natural		thrombin/CaCl_2_	cell adhesive sites	(5, 38, 39)
				pro-angiogenic properties	
				binding sites for myogenic factors (bFGF-2, IGF-1, VEGF)	
dECM hydrogel	natural	*T* = 37 °C	cross-linking methods depending on the hydrogel functionalization (e.g., UV light irradiation in case of methacrylation process)	recapitulation of biological and physical properties of tissue-specific native ECM	(41)
				highly biodegradability	
alginate	natural		CaCl_2,_ MgCl_2,_ SrCl_2,_ BaCl_2_	instantaneous cross-linking in mild condition	(42−44)
				functionalization with RGD-motifs	
				nonbiodegradable	
PEG	synthetic		cross-linking methods depending on the hydrogel functionalization (e.g., UV light irradiation in case of diacrylation process)	easily functionalized with the addition of groups acrylate, thiol, vinyl sulfone, amine, and carboxyl	(36, 47)
				functionalization with proteins and peptides	
PAAm	synthetic		metal ions (Al^3+^, Cr^3+^), organic systems (phenol-formaldehyde), *N*-methylenebis(acrylamide)	functionalization with proteins and peptides	(48, 50, 58)
				tunable mechanical properties by changing the ratio of acrylamide to bis-acrylamide components in the copolymer	
PEGDA	synthetic		UV light irradiation in the presence of photoinitiator	functionalization with proteins and peptides	(51−53)
				low degradation rate and immunogenicity	
				tunable mechanical properties by changing molecular weight/concentration of the polymer	
GelMA	semisynthetic	*T* < 30–35 °C	UV light irradiation in the presence of photoinitiator	retainment of mostly cell adhesive sites from gelatin	(54−56)
				tunable mechanical properties and porosity according to the degree of methacrylation and cross-linking condition	

### 2.1 Most Common Hydrogels for SMTE

Hydrogels are generally classified into two main categories based on their source of origin: naturally derived and synthetic hydrogels.^27^ Naturally derived hydrogels are characterized by high biocompatibility, biodegradability, and low inflammatory response.^28,29^ In particular, hydrogels obtained from the extracellular matrix (ECM) are enriched with a variety of bioactive motifs, including the popular arginine-glycine-aspartic acid (RGD), which promotes cell adhesion and growth.^30,31^ For such biological features, ECM-derived hydrogels enable the recreation of a cell-supportive physiological-like microenvironment suitable for skeletal muscle tissue development. Among ECM-derived hydrogels, collagen, gelatin, and fibrinogen are the most used in SMTE. Collagen is a fibrous protein and is the main component of the ECM, accounting for 25–35% of protein contents in the body.^31−33^ The most used form of collagen is type I, and it can be obtained from various tissues, including skin, ligament, cartilage, and bone, through the use of enzymatic and acid/base processes. Collagen-based hydrogels are formed at physiological temperature (i.e., 37 °C), inducing the assembly of solubilized type I collagen fibrils.^33^ Gelatin is obtained from collagen through a hydrolysis process.^31,34^ As a collagen derivative, gelatin retains the cell-binding sites. Moreover, gelatin possesses reduced immunogenicity potential compared to collagen due to its lower number of aromatic groups, which are removed during the denaturation process.^35,36^ Gelatin owns unique thermoresponsive properties to form physically cross-linked hydrogels at a temperature below 30–35 °C and dissolves as a single coil at a physiological temperature. To further increase the mechanical stability of the resulting hydrogels, collagen and gelatin can be covalently cross-linked with various kinds of cross-linking agents such as transglutaminase or genipin. Fibrin is a branched and microfibrillar polymer formed from fibrinogen by thrombin-catalyzed enzymatic polymerization.^27,37^ Fibrinogen is isolated from blood plasma using precipitation techniques such as cryo-precipitation and ammonium sulfate precipitation.^38^ Since it can be obtained directly from the patient’s blood, fibrinogen holds the potential to be employed as an autologous source, mitigating and evenly eliminating the risks of immunological incompatibility, which usually result when nonautologous sources are used.^28^ In addition to possessing a high abundance of cell attachment sites, fibrin has intrinsic angiogenic properties of paramount importance to promote the vascularization of skeletal muscle constructs upon *in vivo* implantation.^39^ Besides, fibrin hydrogels provide binding sites for growth factors that may augment myogenesis (e.g., basic fibroblast growth factor-2 (bFGF-2) and insulin-like growth factor-1 (IGF-1)).^5^ To further increase the biomimetic potential, hydrogels can also be obtained by decellularizing skeletal muscle’s ECM.^40^ Hence, structural, chemical, and biological complexities of the native microenvironment can be reproduced, thus offering excellent myogenic cues for muscle development.^41^ In addition to ECM-like hydrogels, also naturally derived polysaccharide hydrogels are often employed in SMTE. The most popular is alginic acid (i.e., alginate), a copolymer of (1,4)-linked β-d-mannuronic (block M unit) and α-l-guluronic (block G unit), which can be obtained from various species of brown seaweeds.^42^ One of the main properties of alginate is the ability to undergo instantaneous gelation in the presence of positively charged divalent cations (e.g., Ca^2+^, Ba^2+^, Mg^2+^).^43^ One of the main drawbacks of alginate is the lack of cell-supportive binding sites and its complete inertness. To overcome such an issue, it can be easily functionalized with short peptides containing cell adhesive motifs such as RGD sequences.^44^ Although natural hydrogels are the preferential choice to guarantee a cell-compatible microenvironment, they are limited in terms of precise control over their properties, processability, and biofunctionality.^45^ Unlike their naturally derived counterparts, synthetic hydrogels provide better tunability in terms of mechanical and physical properties.^20^ For instance, viscoelasticity, elastic modulus, permeability, and degradability can be better controlled by precisely adjusting/designing the weight percent, molecular chain length, and cross-linking density.^27,46^ Moreover, synthetic hydrogels tend to have a relatively lower risk of pathogen transfection and batch-to-batch variability.^36^ Among others, typical synthetic hydrogels used for SMTE are based on poly(ethylene glycol) (PEG) and polyacrylamide (PAAm).^47−50^ Due to their high chain mobility and hydrophilicity, they are inherently resistant to protein adsorption. However, they can be covalently modified with short peptides or proteins to favor cell adhesion.^27,46^ Both natural and synthetic hydrogels own the ability to be chemically modified to undergo photo-cross-linking (in the presence of a photoinitiator) to obtain hydrogels for SMTE. For instance, poly(ethylene glycol) diacrylate (PEGDA) is a PEG derivative, which contains double acrylate groups at both ends of the PEG chains.^51−53^ Another typical photo-cross-linkable hydrogel is gelatin methacryloyl (GelMA), obtained by the reaction of methacrylic anhydride (MA) with gelatin.^54,55^ Interestingly, during the chemical modification process, less than 5% of the gelatin amino acid residues are involved, thus preserving the original cell adhesive properties of gelatin.^56,57^

### 2.2 Biochemical Properties

As previously mentioned, ECM-like hydrogels (i.e., collagen, gelatin, fibrinogen) offer cell-supportive features that provide suitable biochemical and biological cues for proper skeletal muscle development. As an alternative to those types of hydrogels, the functionalization of bioinert and synthetic hydrogels, which generally lack cell-adhesives binding sites, can be applied by conjugating or entrapping bioactive biomolecules or peptides into the hydrogel network.^59^ Hence, the attractive features of bioinert hydrogels can be coupled with biologically and biochemically active properties that may further foster myogenic proliferation, viability, and functionality.^59^ For instance, Salimath et al. demonstrated that the RGD functionalization of synthetic PEG hydrogels promoted cell attachment, proliferation, and differentiation, thus forming multinucleated and differentiated myotubes.^60^ In another work, the conjugation of alginate with heparin (i.e., a biomolecule used for growth factors retention) combined with skeletal muscle decellularized ECM (dECM) significantly enhanced cell differentiation and myotube formation in humans skeletal muscle progenitor cells (hSMPCs) compared to individual substrata (i.e., alginate, and dECM).^61^ In a parallel fashion, hyaluronic acid hydrogels conjugated with chondroitin sulfate (i.e., important components of skeletal muscle tissue ECM) were found to support myoblast differentiation and proper integration with the surrounding host tissue upon 4-week implantation.^62^

Besides bioactive molecules, specific myogenesis-inducing growth factors can also be used as an interesting biofunctionalization approach in SMTE. These biological signaling peptides can be linked to the hydrogel matrix through several methods (e.g., covalent bonding, physical entrapment) according to the physicochemical properties of both growth factor and substrate.^9,63^ For instance, the most used myogenic-inducing growth factors are IGF-1, bFGF-2, and vascular endothelial growth factor (VEGF).^64^

Embedding those bioactive molecules into the hydrogel matrix can positively affect muscle cell precursors in *in vitro* conditions by providing supplements to improve proliferation, adhesion, and differentiation.^65,66^ However, their most popular use is associated with *in vivo* application upon implantation directly into the injury site. The therapeutic release of growth factors, which is often combined with muscle cells precursors, enables the creation of a biochemical microenvironment favorable to support skeletal muscle functional regeneration at the injury site.^9^ In one work, bFGF-2 loaded into alginate/hyaluronic acid hydrogels induced an enhancement in the expression of myogenic regulatory factor-related genes, hypertrophy of muscle fibers, and proliferation of muscle satellite cells in the defect area.^67^ In parallel, implantation of PEGylated fibrin gel encapsulating IGF-I induced the restoration of the contractile muscle function and improved the maximal force recovery.^68^ Hydrogels can also be combined with specific growth factors to promote the activation of neighboring tissues (i.e., vascular and nervous tissue) and accelerate the skeletal muscle tissue regeneration process along with host tissue integration. For instance, Sheiki et al. combined GelMA scaffolds with VEGF.^69^ Upon implantation, the controlled release of VEGF induced a functional muscle recovery, an increase in the vascularization, and the anabolic response compared to the untreated control. Similarly, Shvartsman et al. implanted alginate-based hydrogels loaded with VEGF obtaining a significant enhancement in the skeletal muscle innervation and regrowth of damaged nerve axons in the injury site.^70^

### 2.3 Mechanical Properties

The mechanical properties of biomaterials play a fundamental role in SMTE. Among them, substrate stiffness enables the activation of the intracellular signaling process (i.e., mechanotransduction) that can influence critical functions of muscle precursor cells.^64^ Hence, mimicking the stiffness of the native skeletal muscle tissue (Young’s modulus ranging from 10 to 17 kPa) is important for developing a functionally engineered construct.^71,72^ Several studies demonstrated the superior formation of functional myotubes and enhancement in muscle precursor cell differentiation when cultured on substrates whose stiffness matched that of the native tissue.^73^ In this frame, hydrogels own unique physical properties that allow mimicry of the mechanical nature of several soft tissues, including the skeletal muscle. Furthermore, hydrogel mechanical properties can be easily tuned by changing fabrication parameters, such as cross-linking agents and time, as well as hydrogel concentration. Such interesting features give the opportunity to design hydrogels in a wide range of stiffness values. Consequently, optimal fabrication conditions and parameters for proper skeletal muscle development can be selected. For instance, Costantini et al. modulated the compressive modulus properties of GelMA hydrogels by tuning hydrogel concentration.^74^ In particular, the use of higher hydrogel concentrations (6–8% w/v) produced higher compressive modulus (5–10 kPa) compared to those (1–2 kPa) obtained for lower hydrogel concentration (3–4% w/v). Such differences in the hydrogel stiffness were found to have a crucial impact on regulating the behavior of C2C12 myoblasts embedded into the scaffolds. At day 14 of culture, C2C12 myoblasts embedded into low-concentration hydrogels displayed a remarkable amount of myotubes. Contrarily, those encapsulated into higher hydrogel concentrations showed a significant detriment in myogenic differentiation, with minor myotube formation, generally localized in clusters. Such findings might be in contrast with the mechanical properties required for the development of the skeletal muscle tissue. However, it can be explained by the fact that softer mechanical properties are related to a less dense hydrogel matrix that in turn can favor the fusion of myoblasts with adjacent cells and the activation of the metabolic pathways for matrix metalloproteinases. Additionally, the diffusion rate of metabolites and wastes is inversely proportional to the matrix stiffness. Therefore, this might further hamper C2C12 myoblast differentiation within stiffer hydrogels. Similarly, the compressive modulus of GelMA hydrogels was tuned by changing UV-photopolymerization time.^72^ Specifically, cross-linking times of 15, 30, and 60 s enabled the production of hydrogels with a stiffness of 9, 13, and 43 kPa, respectively. As a result, the optimal cross-linking time condition (30 s) allowed closer recapitulation of the mechanical properties of the native muscle tissue.

### 2.4 Electroconductive Properties

Electrical signals play an essential role in cell communication and cellular behaviors (e.g., proliferation, differentiation, tissue maturation), which are critical mechanisms for the functionality and the development of excitable biological tissue. Hence, SMTE encouraged the development of electrically conductive hydrogels to provide engineered platforms that can simultaneously guarantee a tissue-like microenvironment and an efficient delivery of electrical signals.^75,76^ A common approach to develop conductive hydrogels consists in combing the pristine material with conductive polymers such as polyaniline (PANi) or poly(3,4-ethylene dioxythiophene) (PEDOT).^77,78^ For example, Hosseinzadeh et al. interpenetrated a PAAm hydrogel with polyaniline (PANi) as a conductive component.^48^ Compared to pristine hydrogels, satellite cells seeded with the electroconductive samples exhibited a higher degree of differentiation, as remarked by the expression of M-cadherin, a surface molecular marker that exhibited a peak expression in terminal muscle differentiation. Alternatively, conductive hydrogels can also be obtained by including electrical fillers into the hydrogel network. Among them, graphene and its derivatives, such as graphene oxide (GO) and reduced GO (rGO), metal nanowires, and carbon nanotubes (CNTs) have been extensively employed in SMTE.^75,79^ For instance, Jo et al. introduced rGO into PAAm hydrogels to obtain electroconductive hydrogels.^80^*In vitro* studies conducted on C2C12 myoblasts revealed that the presence of rGO significantly enhanced cell proliferation and myogenic differentiation compared to PAAm pristine hydrogels. Moreover, electrical stimulation of C2C12 myoblasts cultured on rGO/PAAm hydrogels for 7 days promoted myogenic gene expression. In another work, CNTs were embedded into GelMA hydrogels and patterned in a uniaxial configuration through the dielectrophoresis (DEP) technique.^81^ Anisotropically aligned GelMA-CNT hydrogels showed higher conductivity than randomly distributed CNTs and pristine GelMA hydrogels. As a result, C2C12 myoblasts encapsulated into aligned GelMA/CNT hydrogels exhibited enhanced maturation and contractile function. Similarly, metallic-submicron glass embedded into GelMA hydrogels increased the electrical conductivity compared to pristine substrates.^82^ Consequently, electroconductive hydrogels have been demonstrated to be more favorable in regulating the adhesion, spreading, and differentiation of muscle precursor cells.

## 3 Hydrogel-Based Methods for Myoblast Alignment

Bulk hydrogels for scaffold-based SMTE were found to successfully provide muscle cell viability, adhesion, and proliferation.^83^ However, due to the lack of specific anisotropy architecture, myoblasts tend to proliferate in entangled and disordered layouts, thus hindering the recreation of a functional skeletal muscle tissue construct.^7,13^ To confer suitable geometrical confinement, which may ultimately result in the generation of a proper cell alignment, first attempts to engineer hydrogels relied on the use of microgrooved hydrogel substrates and micropillars.

### 3.1 Microgrooved Hydrogels

**Figure 1:**
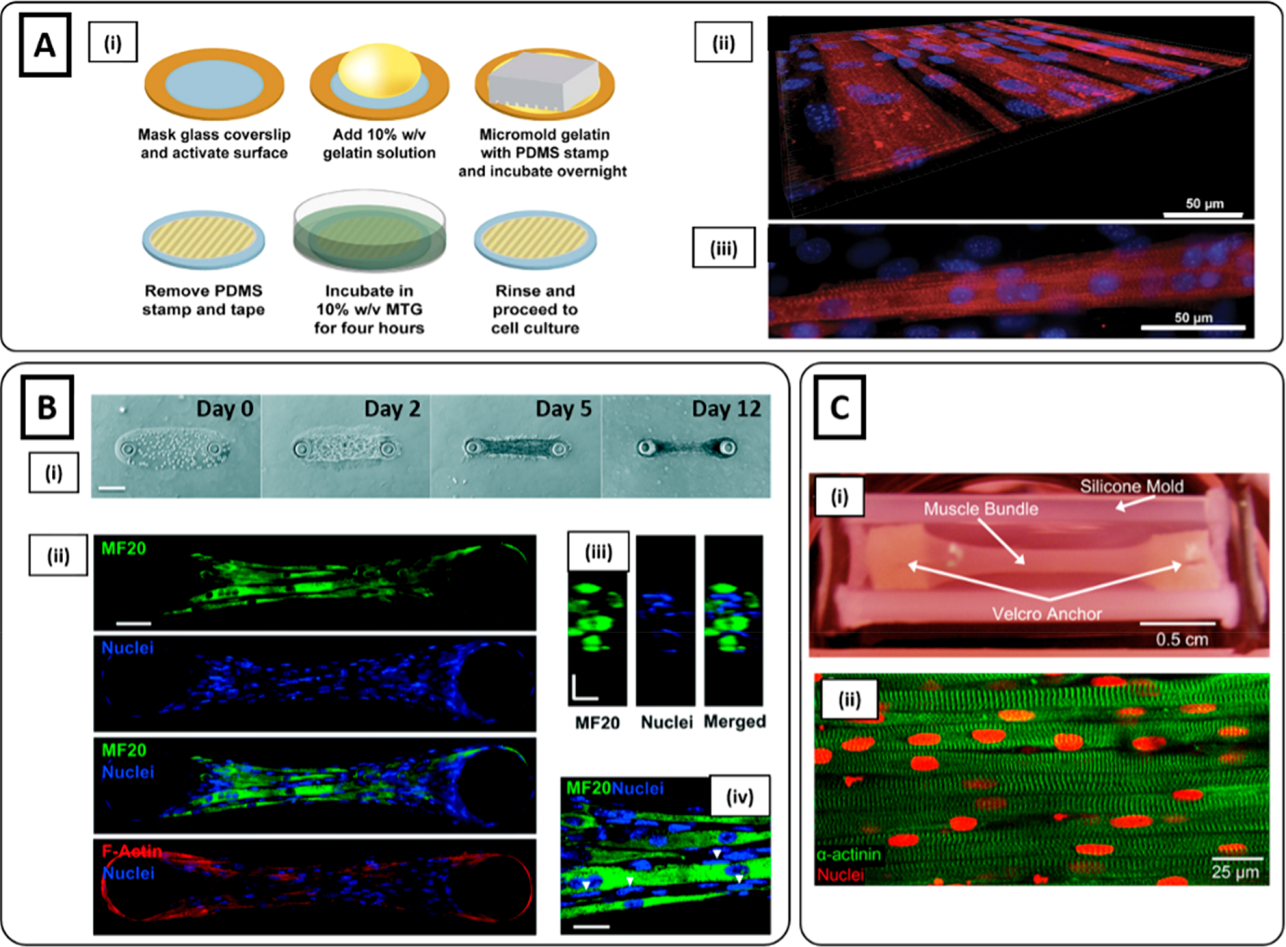
Microgrooved hydrogel and pillar methods for myoblast alignment. (**A**i) Fabrication process of gelatin microgrooved substrate. (ii) Myotubes cultured on micromolded gelatin hydrogels, showing the tissue as a flat monolayer. (iii) Myotubes with visible sarcomeres after 3 weeks of differentiation: blue nuclei, red sarcomeric α-actinin (Reproduced with permission from ref (34). Copyright 2016 Springer Nature CCBY-NC-ND 4.0). (**B**) Muscle tissue development on GelMA hydrogel anchored around two hydrogel pillars. (i) Brightfield images showing an increase in cell growth, alignment, and compaction as a function of days of culture. (ii) Immunofluorescent staining images at day 12 of myosin heavy chain (MF20) (green), nuclei (blue), and F-actin (red) depicting highly matured muscle tissue. (iii) Cross-sectional image illustrating muscle-like fascicular structure. (iv) High magnification (100×) image depicting the arrangement of nuclei on the periphery of myotubes (white arrows). Scale bars: (i) 150; (ii) 50; (iii and iv) 20 μm (Reproduced with permission from ref (95). Copyright 2017 Royal Society of Chemistry). (**C**i) nSKM-laden fibrin hydrogel in a silicone mold anchored on each end to velcro pillars. (ii) Immunostaining of α-actinin (green) and nuclei (red) at day 14, showing highly aligned multinucleated myotubes with ubiquitous cross-striations (Reproduced with permission from ref (96). Copyright 2011 Elsevier).

One of the earliest attempts to investigate myoblast alignment through hydrogel engineering relies on the fabrication of substrates patterned with microgrooves obtained through micromolding methods (see section 4.1).^50,84,85^ Generally, microgrooves have a width lower than 10 μm to reproduce cellular focal contacts.^86^ Once cells are seeded on the micropatterned structure, their myoblast behavior is regulated by contact guidance phenomena, which are defined as a class of processes involving cellular contraction-mediated morphogenesis under boundary constraints. Thus, the geometrical confinement assists the myoblasts’ behavior, which tend to align along the microgroove direction to form muscle-like structures.^19,86,87^ Bettadapur et al. employed a micromolding technique to fabricate gelatin-based microgrooved structures as a substrate to culture C2C12 myoblasts (Figure 1Ai).^34^ Muscle cells aligned along the microgroove direction and multinucleated myotubes were obtained after 3 weeks of culture (Figure 1Aii, iii). Microgrooves can also be employed as a platform to study the effect of different width dimensions on cell alignment behavior. For instance, Hosseini et al. fabricated a GelMA-based micropatterned substrate with two different groove sizes (i.e., 50 and 100 μm, respectively). As a result, it was observed that smaller grooves induce higher cell alignment compared to wider ones. In addition to the width, also the depth of such microgrooves can be tailored. In particular, larger channels can be produced to accommodate a higher cell volume, enabling the recreation of a 3D microenvironment. For example, Hume et al. patterned PEG hydrogels to create 3D channels with different depth dimensions (i.e., 100 and 200 μm, respectively).^88^ It was found that deeper channels promote cell proliferation and multilayer cell culture, which in turn provided an improvement in cell alignment.

### 3.2 Pillars

Alternatively, micropillars can also be used to engineer hydrogels toward cell alignment. Such an approach is designed to reproduce the *in vivo* musculoskeletal microarrangement, characterized by force transmission from muscle to bone through a connecting tendon.^3,89,90^ Generally, the hydrogel solution is cast directly on micropillars or in a mold anchored by micropillars.^91,92^ When myoblasts start to exert an isotropic contractile force on the hydrogel, the anchored micropillars generate a passive longitudinal tension, restricting the cell compaction process along the longitudinal direction.^86,93,94^ Agrawal et al. assessed the efficacy of such method, comparing cell alignment and maturation of C2C12-laden GelMA anchored onto two pillars against a free-form hydrogel configuration.^95^ Cells cultured in unanchored hydrogels collapsed inward and formed cell agglomerates. Conversely, cells cultured in the presence of pillars showed an evident alignment by day 2, followed by the formation of a dense construct at day 5 and further compaction of muscle bundles by day 12 (Figure 1Bi). Moreover, as confirmed from immunofluorescence images, myoblasts formed differentiated myotubes with a peripheral nuclei arrangement (Figure 1Bii–iv). Similarly, Hinds et al. encapsulated rat neonatal skeletal myoblasts (nSKM) in a fibrinogen-based solution.^96^ The cell-laden solution was cast in a silicone mold anchored by two velcro pillars (Figure 1Ci). After 2 weeks, a highly aligned and fully striated construct was obtained (Figure 1Cii). In addition to acting as boundary conditions, the micropillars may also serve as a tool to quantify the force generated by muscle cells by measuring pillar displacements in response to muscle contractions over time. Cvetkovic et al. employed a stereolithography (SLA) 3D printing apparatus to fabricate a millimeter-scale hydrogel device composed of two flexible pillars connected by a compliant beam.^97^ C2C12 myoblasts were mixed with fibrinogen-based hydrogel solution and dispensed around the pillars. After 2 weeks of differentiation, aligned and cross-striated myotubes were obtained. Moreover, by using a viscoelasticity model, it was possible to convert the pillars deflection into the active force generated by the muscle strip in response to electrical stimulation.

## 4 Hydrogel-Based Fiber Biofabrication Methods

**Figure 2:**
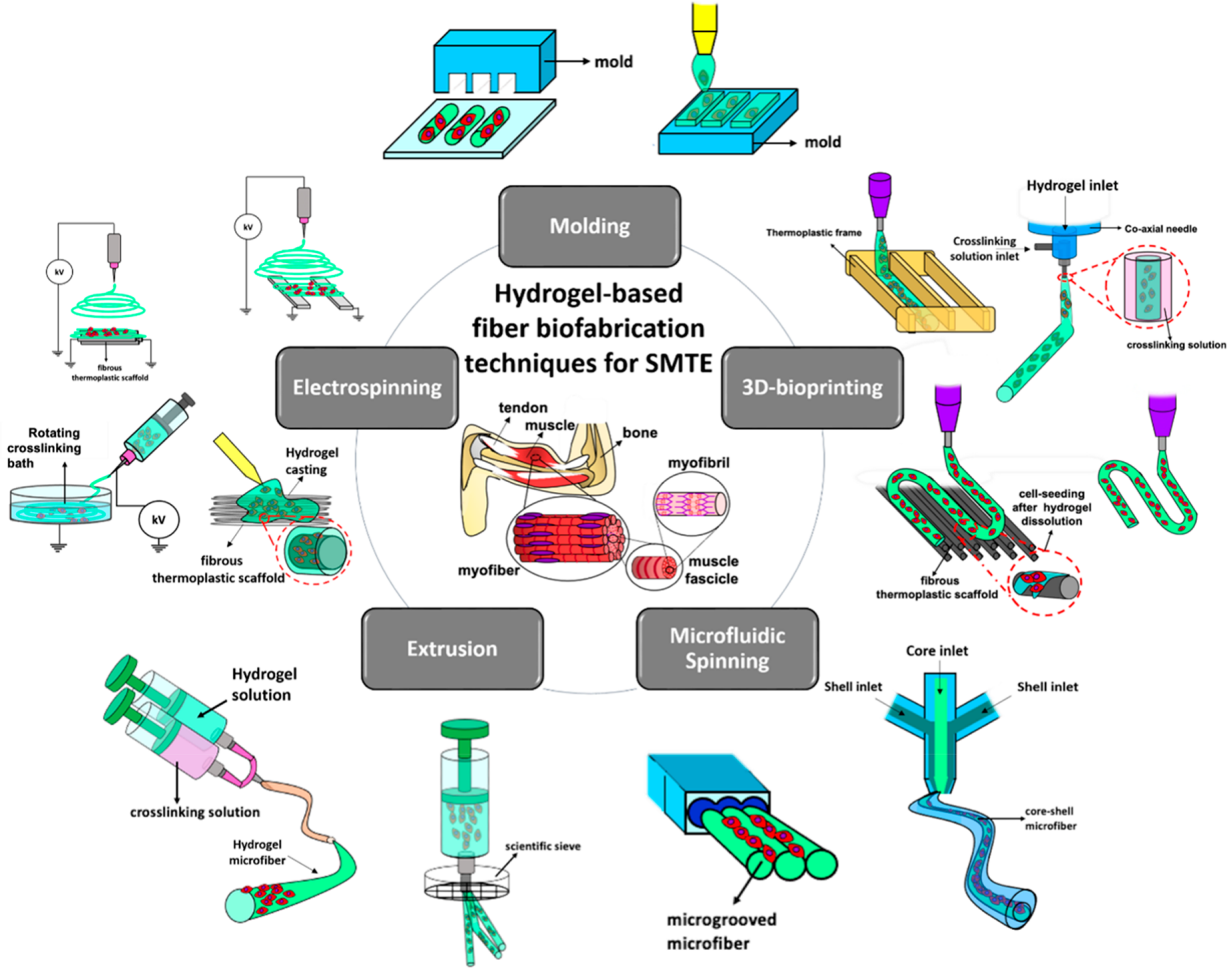
Schematic representation of hydrogel-based fiber biofabrication techniques (i.e., molding, electrospinning, 3D bioprinting, extrusion, and microfluidic spinning) and their specific subcategories, used for the development of advanced skeletal muscle tissue constructs.

Although both microgrooved surfaces and micropillars provide remarkable platforms to elucidate the basic mechanism of myoblast alignment and enable the generation of muscle-like structures, such methods present some limitations. For instance, microgrooved hydrogels generally recreate 2D platforms, which fail in guaranteeing the 3D cues necessary to build a realistic physiological-like microenvironment. Moreover, pillar-based technologies hardly allow the control over the scaffold geometry, which plays a cardinal role in myoblast alignment and tissue development. Furthermore, such approaches lack scalability, which is fundamental for the recreation of full-scale skeletal muscle constructs. In light of this, hydrogel microfibers constitute an ideal platform for muscle cell elongation and alignment along cylindrical-shaped biomimetic scaffolds. Moreover, fiber diameters can be properly tailored to guarantee an effective muscle precursors cells alignment. Methods for the biofabrication of SMTE hydrogel-based microfibers are diverse and include (i) molding, (ii) electrospinning, (iii) 3D bioprinting, (iv) extrusion, and (v) microfluidic spinning (Figure 2, Table 2). In the following text, each paragraph (except for section 4.3) is divided into *cell-seeded* and *cell-laden* hydrogel fiber-based biofabrication techniques according to whether the fabrication parameters (e.g., solvent used, cross-linking condition, viscosity, high voltage, pressure, temperature) are compatible with cell encapsulation methods.

**Table 2:** Hydrogel Fiber-Based Biofabrication Methods for SMTE

hydrogel-based fiber biofabrication technique	hydrogel	muscle cells	cell-seeded/cell-laden	external stimulation	fiber diameter	*in vitro*/*in vivo* main outcomes	ref
molding	alginate/fibrinogen	C2C12	cell-seeded		∼20 μm	freestanding hydrogel fibers	(103)
						C2C12 myoblasts remained adhered after the dissolution of the sacrificial layer used as a substrate	
						C2C12 myoblasts aligned along the microfiber direction after 3 days of culture	
molding	GelMA	C2C12	cell-laden	mechanical stretching	∼400 μm	formation of 10 cm-long microfibers	(72)
						differentiated myotube after static unaxial mechanical stimulation (35% strain)	
direct electrospinning	alginate/PEO/fibrinogen	C2C12	cell-seeded	mechanical stretching	∼10 μm	aligned hydrogel microfibers bundle mimicking muscle structure	(112)
						densely aligned MHC-positive myotubes after uniaxial mechanical stimulation (static and cyclic)	
hybrid electrospinning	alginate/PEO/	C2C12	cell-seeded		∼0.2 μm	fabrication of a hierarchical scaffold with hydrogel nanofibers deposited onto a PCL structure	(120)
						generation of topographical cues obtained by leaching process	
						aligned and differentiated myotubes after 21 days of culture	
direct electrospinning	alginate/PEO	C2C12	cell-laden		∼60 μm	defined and bead-less hydrogel electrospun microfiber with high cell viability (>80%)	(122)
						elongated and differentiated myoblasts after 7 days of culture	
hydrogel casting on polymeric nanofibers	alginate/gelMA	C2C12	cell-laden		∼400 μm (core)	generation of composite core–shell microfibers	(128)
					∼200 μm (hydrogel thickness)	C2C12 myoblasts homogeneously distributed and aligned along microfiber direction after 2 days of culture	
						improved electroconductivity and enhanced myogenic gene expression in microfibers coating with rGO	
indirect 3D bioprinting	fibrinogen/gelatin/hyaluronic acid	hMPC	cell-laden		∼300 μm	82% of functional skeletal muscle recovery after 8 weeks of *in vivo* implantation in TA defect of a rodent model	(145)
						regeneration of highly organized muscle structure in the defect site	
						innervation and vascularization *in vivo*	
microfluidic-assisted 3D bioprinting	monoacrylated-PEG fibrinogen/alginate	C2C12	cell-laden		∼250 μm	high-resolution 3D bioprinted cell-laden hydrogel filaments	(7)
						formation of completely striated myofibers exhibiting spontaneous contraction	
						formation of an organized and mature muscle-like structure after 28 days of *in vivo* implantation	
direct 3D bioprinting	collagen	C2C12	cell-laden	electrical	∼350 μm	alignment of GNWs embedded into collagen-bioink using optimal 3D printing pressure and nozzle moving speed	(197)
						alignment of C2C12 myoblasts along the printing direction	
						enhancement in cell alignment and MHC expression after electrical stimulation	
hybrid 3D bioprinting	alginate/PEO	C2C12	cell-laden			homogeneous cell release onto thermoplastic 3D printed structure	(159)
						generation of a cylindrical bundle-like structure obtained by rolling the 3D printed scaffold	
						cell alignment along the microfiber longitudinal direction	
extrusion	fibrinogen	C2C12	cell-seeded		∼60–80 μm	aligned superficial microgrooves obtained by MES-based chemical treatments	(165)
						cell alignment along the microgroove direction	
extrusion	GelMA/PEGMA	C2C12	cell-laden	mechanical stretching	∼100−300 μm	fabrication of microfiber with different diameter by changing sieve pore size	(167)
						high cells viability (<90%)	
						MHC-positive myotubes under static mechanical stimulation	
microfluidic spinning	GelMA	C2C12	cell-seeded		∼500 μm	fabrication of microgrooved microfibers	(186)
						C2C12 myoblasts alignment along the microgrooves after 3 days of culture	
microfluidic spinning	alginate/collagen	C2C12	cell-laden	mechanical stretching	∼150 μm	differentiated C2C12 myoblasts after 2 days of cyclic mechanical stretching	(195)

### 4.1 Hydrogel Micromolding

Micromolding is a facile, cost-effective, and intuitive microfabrication technique to fabricate hydrogel microfibers.^21,98,99^ It is based on the use of a mold applied to a hydrogel precursor, which is subsequently cross-linked to assume the negative shape of the mold.^19^ Polydimethylsiloxane (PDMS) is the most commonly used material for the fabrication of molds thanks to its tunable mechanical strength and elasticity, transparency, biocompatibility, and high fidelity of molding micro- and nanostructural features.^19,37,100^ Moreover, PDMS possesses a hydrophobic surface, which may facilitate mold detachment from cross-linked hydrogels.^100^ In addition to PDMS, poly(methyl methacrylate) (PMMA), silicone, polytetrafluoroethylene (PTFE), glass, and metals are also frequently used to produce micromolds.^19,37,86,101^ Besides stiff templates, soft, cell-compatible, and dissolvable materials can also be employed by acting as sacrificial molds.^37,102^ Micromolded hydrogels can be cross-linked by different methods, including UV-photopolymerization, physical, and thermal cross-linking.

#### 4.1.1 Cell-Seeded Molding

**Figure 3:**
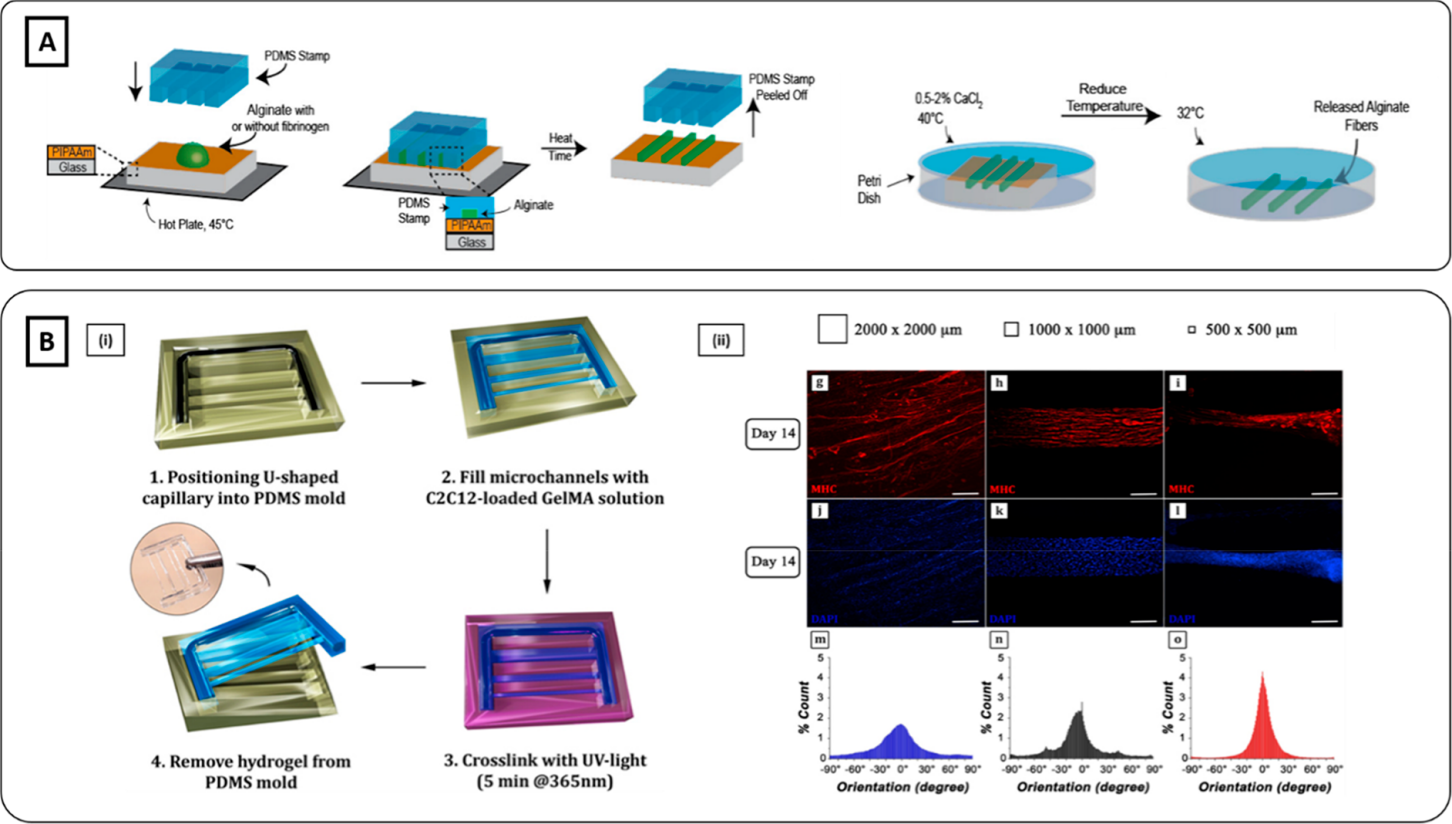
Hydrogel molding methods. (**A**) Schematics of alginate-based microfibers fabrication using hydrogel molding methods (Reproduced with permission from ref (103). Copyright 2014 IOP publishing). (**B**i) Schematics of C2C12-laden GelMA fibrous scaffold micromolded into a U-shape mold. (ii) Smallest and middle cross-section channels (0.5 mm × 0.5 mm and 1 mm × 1 mm, respectively) induced higher cell alignment and compaction compared to the largest one (2 mm × 2 mm). Scale bar 100 μm (Reproduced with permission from ref (74). Copyright 2017 The Authors, Frontiers CCBY-NC-ND 4.0).

Microfibers can be easily fabricated by casting a hydrogel solution on a substrate and molding it with a grooved stamp. Moreover, by depositing the hydrogel solution on a sacrificial substrate, free-standing fibers can be produced. Szymanski et al. cast alginate/fibrinogen hydrogel solution on a coverslip coated with sacrificial poly(*N*-isopropylacrylamide) (PIPAAm), heated to 50 °C to prevent PIPAAm dissolution, and then micromolded by using a PDMS mold.^103^ Alginate/fibrinogen microfibers were dried, detached from PDMS, and then cross-linked with a mixture of CaCl_2_/thrombin solution. Freestanding hydrogel fibers were obtained by the dissolution of the surface obtained by cooling the temperature at PIPAAm dissolution critical value (i.e., ∼32 °C) (Figure 3A). Before detaching, C2C12 myoblasts were seeded and cultured for 12 h to allow cell adhesion. Fluorescent images of cell nuclei and actin filaments confirmed that cells remained adhered to microfibers surface after the thermally triggered release. Moreover, at day 3 of culture, cells aligned along the fiber axis direction. To improve the biochemical cues, the surface of alginate/fibrinogen hydrogel microfibers was further functionalized by performing microcontact printing. To this aim, multiple ECM protein types such as fibronectin and laminin were employed.^104^ By day 7, cells showed uniaxial alignment over the alginate/fibrinogen microfibers. Interestingly, it was observed that cells generated a contractile force, thus pulling microfibers around themselves and forming hollow, tube-like structures. Hence, such constructs mimicked the basal lamina surrounding myofibers present in *in vivo* conditions. To successfully recapitulate the structure of a muscle bundle, single fibers were parallelly tethered on a PDMS square frame and cultured for 3 days. Once the PDMS frame was lifted out of culture medium, the capillary forces induced fiber bundling and the formation of muscle-like fascicles. Confocal imaging showed that seeded C2C12 myoblasts remained viable, highly aligned, and surrounded by the microfiber.

#### 4.1.2 Cell-Laden Molding

Micromolding approach can be easily adapted for the fabrication of cell-laden hydrogel fiber-shaped constructs. Microfibers can be obtained by injecting hydrogel/cells solution in molds with different geometries such as cylindrical or channel-like.^5,72^ Moreover, stamp design configurations and dimensions can be adapted in order to produce fibers with different diameters and study the effect of geometrical confinement in muscle cell orientation. For instance, Costantini et al. employed three PDMS molds formed by microchannels with different cross sections dimensions (e.g., 2 mm × 2 mm, 1 mm × 1 mm, 0.5 mm × 0.5 mm).^74^ GelMA/C2C12 solution was cast on the mold and cell-laden microfibers were formed upon UV-photopolymerization (Figure 3Bi). After 2 weeks of culture, immunofluorescence images revealed higher myotube compaction and enhanced parallel orientation in microfibers with the smallest and middle cross-section compared to the largest one (Figure 3Bii).

### 4.2 Hydrogel Electrospinning

Electrospinning is a relatively old fashion technique widely employed in SMTE for the ability to produce nanofibers able to mimic the scale size of the proteins constituting the ECM microenvironment.^12^ In a traditional electrospinning fabrication process, the spinning solution is first pumped out by a syringe pump or by pressured gas into the tip of a needle (e.g., spinneret) connected to a high direct current (DC) voltage source. The solution, which initially forms a hemisphere drop due to the surface tension, is charged and elongated into a solution jet under the increasing high-voltage power and then directed toward the grounded collector.^16,25^ According to the type of collector employed, nanofibers are deposited to obtain a fibrous mat with a random or preferential arrangement.^8^ In SMTE applications, the aligned configuration is always preferred to ensure proper topographical cues. Such anisotropic architecture is obtained by depositing nanofibers on parallelly arranged electrodes or on rotating cylinders.^12^ Due to the high spinnability performances, the selection of biomaterials employed for electrospinning is usually oriented to synthetic polymers, including PCL, polyurethane (PU) and polylactic acid (PLLA).^8,15^ In order to improve the biochemical cues (e.g., cell adhesiveness and hydrophilic properties), synthetic polymers can be combined with naturally derived biomaterials (e.g., collagen, chitosan) or functionalized by specific surface modification treatments (e.g., oxygen plasma treatment).^12^ Alternatively to these strategies, the employment of hydrogels as a biomaterial substrate for electrospinning has recently gained attention.^105^ Hydrogels can be used as a spinning solution or combined with a previously fabricated synthetic fibrous mat to create core–shell composite microfibers. Moreover, advances in biofabrication technologies allow hydrogel electrospinning to be considered as a potential platform for cell encapsulation.^106−110^ All these approaches were explored in the field of SMTE.

#### 4.2.1 Cell-Seeded Direct Electrospinning

**Figure 4:**
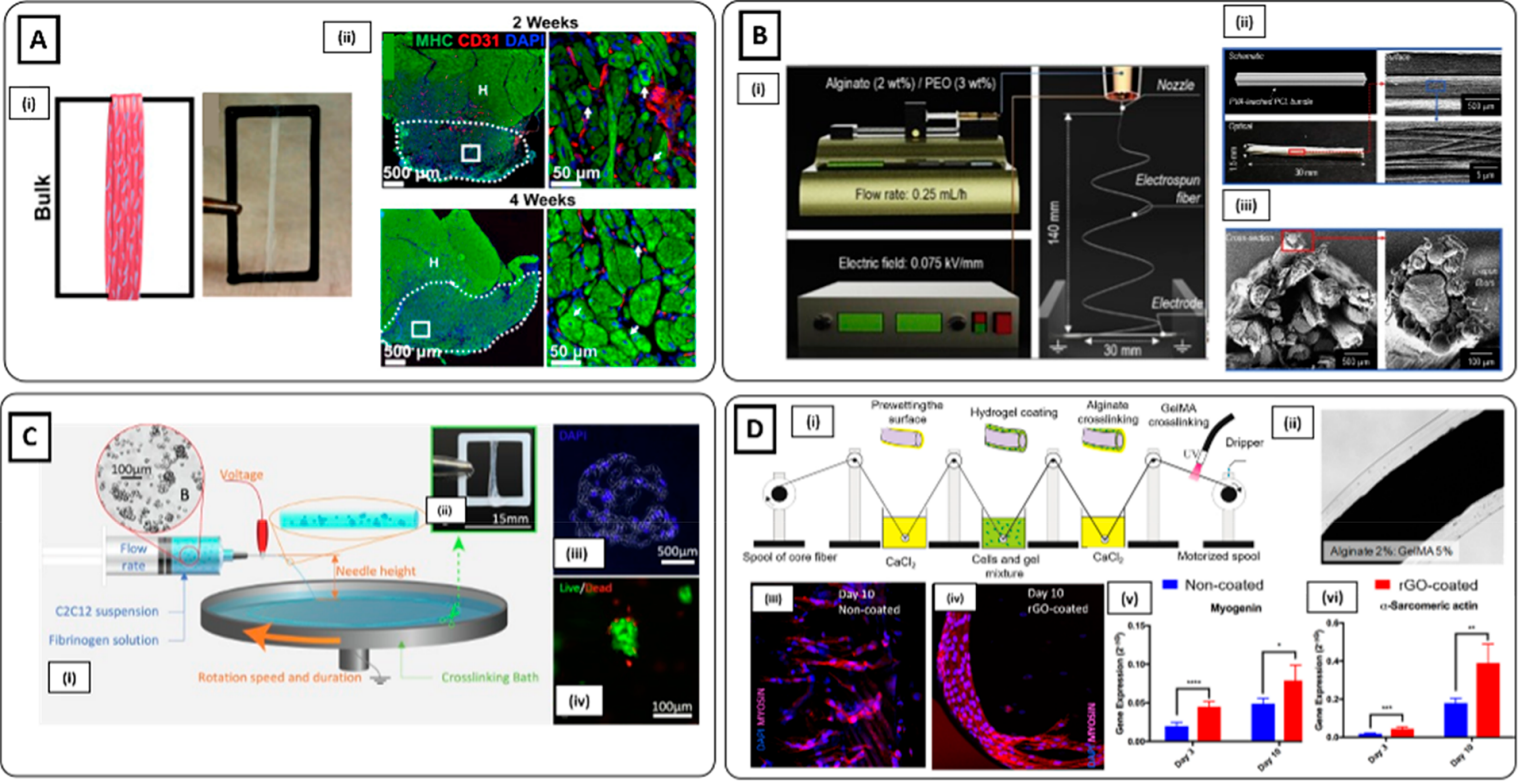
Hydrogel electrospinning methods. (**A**i) C2C12-seeded electrospun fibrinogen bundle collected on ABS frame. (ii) Immunofluorescence staining of MHC (green), CD31 (red), and nuclei (blue) of volumetric muscle loss (VML) defects treated with C2C12-seeded scaffolds at 2 and 4 weeks. High densities of centrally nucleated myofibers and a dense vascularized network were detected (Reproduced with permission from ref (118). Copyright 2018 Elsevier). (**B**i) Schematics of alginate/PEO electrospinning process and (ii and iii) schematic, optical, scanning electron microscopy (SEM) images of the muscle-mimetic electrospun bundle structure (Reproduced with permission from ref (120). Copyright 2019 Elsevier). (**C**i) Schematic representation of cell electrospinning of C2C12 myoblast agglomerates. (ii) Cell-laden scaffold wrapped around an ABS frame. (iii) Cross-section of cell-laden scaffold on day 0 stained with DAPI (blue). (iv) Live (green) and dead (red) staining of cell-laden bundle showing that cell electrospinning process enabled the preservation of cell viability (Reproduced with permission from ref (123). Copyright 2019 Elsevier). (**D**i) Schematic of the reel-to-reel fabrication process of cell-laden composite fibers. (ii) Brightfield image of composite microfiber. (iii and iv) MHC immunostaining (red) and (v and vi) transcript levels of myogenic markers assessed higher muscle differentiation in rGO-coated composite microfibers after 10 days of culture (Reproduced with permission from ref (128). Copyright 2019 American Chemical Society).

Due to the low electroconductive capacity, a successful hydrogel electrospinning process to produce bead-free nanofibers can be challenging.^16^ For this reason, hydrogel formulations can be combined with electroconductive materials. Besides increasing the hydrogel spinnability potential, such an approach may also be beneficial for SMTE applications by enhancing muscle differentiation and maturation.^77^ From this perspective, Ostrovidov et al. combined gelatin with multiwalled carbon nanotubes (MWNTs) to electrospun electroconductive hybrid nanofibers for skeletal muscle development.^111^ Gelatin-MWNTs nanofibers were deposited on a parallel electrodes array to obtain an anisotropic arrangement. Gelatin fibers without MWNTs were referred to as a control. After 4 days of culture, C2C12-seeded on MWNTs-nanofibers exhibited an enhancement of the myogenin expression and the amplitude of myotube contractions under electrical stimulation compared to the control group. Moreover, the incorporation of MWNTs in the nanofibers induced an increase of the mechanical properties, which was translated in the upregulation of mechanotransduction-related genes (i.e., focal adhesion kinase). Alternatively, the formation of aligned fibrous structure can also be obtained by collecting electrospun hydrogel nanofibers into a grounded circulating cross-linking bath.^112−115^ Moreover, such assembling approach promotes nanofiber stretching, which can be further enhanced by subsequently collecting fibers on a plastic frame.^116,117^ Gilbert-Honick et al. fabricated hydrogel nanofibers by simultaneously extruding in parallel alginate and fibrinogen.^118^ The solutions were mixed through the tip of an in-line double syringe mixing system and collected in a grounded rotating bath containing thrombin/CaCl_2_ solution. The cross-linked fibrin-alginate nanofibers were then soaked in sodium citrate to induce the dissolution of alginate and subsequently wrapped around a custom-made acrylonitrile butadiene styrene (ABS) frame to form aligned fibrous bundles (Figure 4Ai). C2C12 myoblasts seeded on the fibrous bundles formed densely aligned myosin heavy chain (MHC)-positive myotubes with sarcomeric striations. Moreover, C2C12-seeded scaffolds exhibited spontaneous contraction and generated approximately 1 mN of force in response to electrical stimulation. To further investigate the muscle regeneration potential, C2C12-seeded scaffolds performance was also tested *in vivo* by implantation into the tibial anterior (TA) defect of a rat model. After 4 weeks, constructs enabled a remarkable muscle regeneration with a high number of centrally nucleated MHC myofibers, along with a robust and dense capillary network (Figure 4Aii).

#### 4.2.2 Cell-Seeded Hybrid Electrospinning

To increase the overall stability and the mechanical properties of hydrogel-based electrospun scaffolds, nanofibers can be deposited on a thermoplastic supportive structure to create a hierarchical hybrid construct.^119^ For instance, Yeo et al. electrospun alginate/poly(ethylene oxide) (PEO) nanofibers on a micropatterned PCL structure obtained by 3D printing process.^120^ Herein, nanofibers were anisotropically deposited by positioning the PCL structure between parallel-arranged cylindrical electrodes (Figure 4B). Besides contributing to improve the overall stability, PCL was also employed to generate topographical cues by leaching process through the incorporation of a thermoresponsive sacrificial material (i.e., poly(vinyl alcohol) (PVA)). C2C12 myoblasts were seeded on the hierarchical structure and cultured up to 21 days, thus enabling the formation of highly aligned and mature myotubes expressing MHC.

#### 4.2.3 Cell-Laden Electrospinning

Although electrospinning methods can successfully generate nanofibrous scaffolds to guide muscle cell alignment and promote differentiation, the cell seeding procedure may cause a nonhomogeneous cell distribution.^107,121^ Indeed, seeded cells mainly tend to remain on the surface of the scaffolds, thus limiting cell infiltration throughout the thickness of the construct. Cell-electrospinning may offer the possibility to overcome this issue by directly encapsulating cells into hydrogel nanofibers.^106^ However, cell-electrospinning is associated with several shortcomings, mainly due to the cell-unfriendly parameters used for the fabrication process. For instance, high voltage can cause cell membrane rupture, thus often leading to cell death.^109^ Hence, a compromise between electrospinnability and cell viability must be achieved in order to guarantee a successful nanofibers fabrication with viable and functional muscle cells. This requires an adjustment of traditional electrospinning protocols and process parameters used to produce cell-free hydrogel nanofibers. Yeo et al. succeeded in electrospinning high viable and bead-free C2C12-laden alginate/PEO nanofibers by using a relatively low voltage and subsequently adjusting other fabrication parameters (e.g., nozzle-to-electrode distance, electrode-to-electrode distance).^122^ Moreover, cells were differentiated and uniaxially stretched on the longitudinal axis after 7 days of culture. In another work, preservation of cell viability without compromising the electrospinnability was attempted by encapsulating C2C12 myoblasts in fibrinogen/PEO as cellular aggregates.^123^ C2C12 aggregates were successfully extruded into a thrombin/CaCl_2_ rotating bath and resulted in being highly viable (Figure 4C). Moreover, C2C12 myoblasts formed multinucleated myotubes with myogenic expression over 7 days of culture.

#### 4.2.4 Cell-Laden Hydrogel Casting on Thermoplastic Nanofibers

Besides hydrogel electrospinning, hydrogel nano/microfibers can also be generated by indirect approaches relying on the encapsulation of fibrous synthetic constructs, generally obtained by the electrospinning process, in cell-laden or cell-free hydrogels. Once the hydrogel solution undergoes polymerization after being deposited on synthetic nanofibers, a core–shell composite microfiber is formed.^124,125^ Generally, a fibrous structure is characterized by an anisotropic arrangement obtained by uniaxial deposition on parallel electrodes or by assembly through textile-forming technologies (e.g., weaving, reeling).^126^ Among others, one approach used to create such a multicomponent structure consists of directly casting cell-free or cell-laden hydrogel onto the anisotropic fibrous structures. Besides offering a more cell-friendly microenvironment than pristine polymeric networks, hydrogel coatings guarantee improved mechanical properties and the preservation of the original scaffold microstructure by preventing the winding and twinning of aligned fibers. Wang et al. developed a core–shell scaffold combining PCL/silk fibroin/(PANi) nanofibrous aligned yarns obtained by dry-wet electrospinning and a PEG-*co*-poly(glycerol sebacate) (PEGS-M) photocurable hydrogel.^127^ C2C12 myoblasts seeded on the surface of the fibrous yarns were encapsulated into the PEGS-M hydrogel, and a core–shell structure was formed after photopolymerization by UV light irradiation. C2C12 myoblasts formed multinucleated and MHC positive myotubes after 1 week of culture. An alternative method to fabricate composite core–shell microfibers was proposed by Fallahi et al., who successfully developed an automated custom-made device to perform reel-to-reel multistep hydrogel coating of microthreads from a wide range of material (i.e., PLLA, cotton, polydioxanone).^128^ Basically, nanofibers were first soaked through a coagulation bath of CaCl_2_ and subsequently passed to alginate/GelMA/C2C12 myoblasts solution to form a layer of cell-laden prepolymer solution around the synthetic core. Composite microfibers were finally obtained by soaking them in a CaCl_2_ bath and then by UV light irradiation to cross-link alginate and GelMA components, respectively (Figure 4Di, ii). Encapsulated C2C12 myoblasts resulted in being uniformly encapsulated and aligned along the microfibers direction after 2 days of culture. To further improve the muscle regeneration potential, a coating of rGO was deposited on the synthetic nanofibers to increase the electroconductivity. After 10 days, myogenic gene expression was found to be significantly upregulated in C2C12 myoblasts in rGO-coated versus noncoated fibers (Figure 4Diii–vi).

### 4.3 Hydrogel 3D Bioprinting

3D bioprinting is an emerging technology that relies on the layer-by-layer deposition of cell-laden hydrogels in a spatially defined manner.^124,129−132^ Thanks to these promising features, 3D bioprinting is rapidly rising as a potential technique for the fabrication of physiologically relevant 3D scaffolds with complex architectures to reproduce a wide variety of biological tissues, including skeletal muscle.^133−138^ Various 3D bioprinting techniques, such as laser-based or inkjet-based bioprinting, as well as extrusion-based 3D bioprinting, have been employed to recapitulate the native morphology of skeletal muscle tissue at a large scale.^139^ However, among these 3D bioprinting approaches, extrusion-based 3D bioprinting also enables the recreation of the muscle microenvironment on a microscopic scale by extruding a continuous cell-laden hydrogel filament through a nozzle by means of pneumatic or mechanical pressure.^7,12,140^ Such filament is then deposited in a layer-by-layer fashion to rebuild a full-scale muscle construct. To ensure a suitable microenvironment for proper myoblast alignment and differentiation, the hydrogel filament must be highly defined and physically stable. To this aim, several 3D printing strategies are employed: (i) direct, (ii) microfluidic-assisted, and (iii) indirect 3D hydrogel bioprinting. Alternatively, 3D bioprinted hydrogel filaments can be employed as a cell carrier to ease the homogeneous release of muscle cells on aligned fibrous structures (so-called hybrid 3D bioprinting).

#### 4.3.1 Indirect 3D Bioprinting

**Figure 5:**
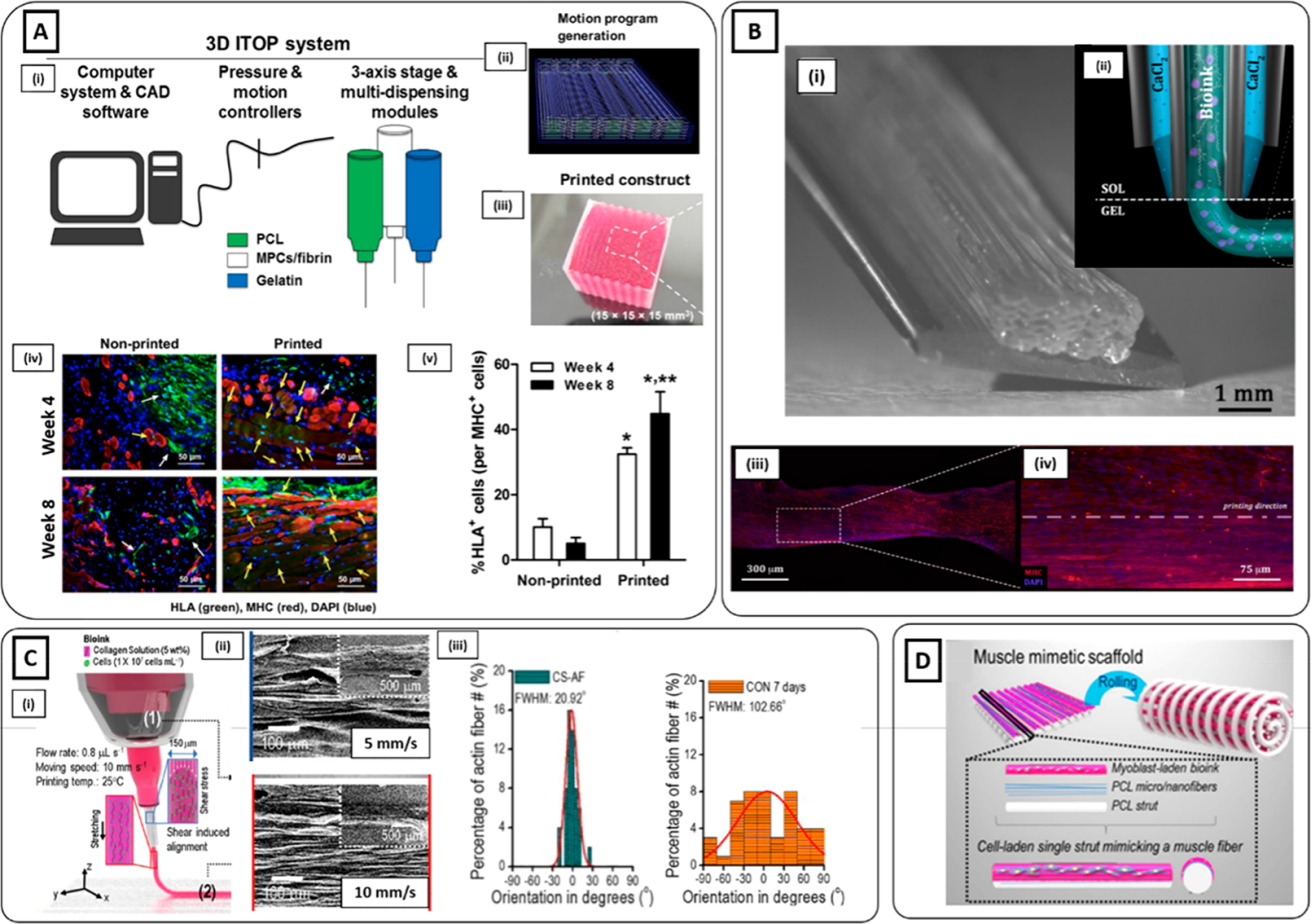
3D Bioprinting methods. (**A**i) Schematic of the 3D bioprinting ITOP system. The motion program (ii) is transferred to the operating computer of ITOP. The cell-laden bioink, the acellular sacrificial hydrogel, and the supporting PCL pillar are loaded in the multidispensing modules, and (iii) the 3D bioprinted construct is generated. (iv) Immunofluorescent staining (human leukocyte antigen (HLA), green; MHC, red; nuclei, blue) of 3D bioprinted and nonprinted scaffolds after 4 and 8 weeks of implantation. (v) Higher numbers of HLA+/MHC+ cells were found in the 3D bioprinted scaffold (Reproduced with permission from ref (145). Copyright 2018 The Authors, Springer Nature CCBY-NC-ND 4.0). (**B**i) High-resolution myoblast-laden scaffold obtained by (ii) coaxial delivery of monoacrylate-PEG fibrinogen/alginate and CaCl_2_. (iii) Immunofluorescent image of MHC (red) and DAPI (blue) and (iv) high magnification of the ROI after 15 days of culture showed highly aligned and differentiated myotubes [(i, iii, iv) Reproduced with permission from ref (7). Copyright 2017 Elsevier CCBY-NC-ND 4.0. (ii) Reproduced with permission from ref (147). Copyright 2016 IOP Publishing]. (**C**i) Schematic image of the 3D bioprinting extrusion process inducing the alignment of collagen fibrils through the application of shear stress. (ii) SEM images of collagen fibrils showing an enhancement in the uniaxial orientation by increasing the nozzle moving speed. (iii) Enhancement in actin filament orientation for samples treated with Gly/KCl (left) compared to those without treatment (right) (Reproduced with permission from ref (157). Copyright 2019 Elsevier). (**D**) Schematics of the 3D hierarchical scaffold obtained by 3D bioprinting myoblast-laden bioink on an electrospun PCL structure. The scaffold was self-rolled to produce a muscle-like bundle structure (Reproduced with permission from ref (159). Copyright 2016 IOP Publishing).

Hydrogel 3D bioprinting can be combined by a supportive frame generally made of thermoplastic polymer (e.g., PCL). The supportive structure is generally coprinted with the cell-laden hydrogel by using a complex 3D printing apparatus able to process multiple biomaterials either toward sequential printing or employing multiple printing heads.^141,142^ The thermoplastic polymer can be deposited parallelly to the hydrogel filament or as an external contour of the printed hydrogel scaffold.^141,143^ Hence, the thermoplastic polymeric frame can support the hydrogel filament during the deposition step, both sustaining the fiber formation before cross-linking and providing mechanical strength to hold the overall structure by avoiding the collapsing over the layer-by-layer deposition.^144^ Besides contributing to the structural integrity of multilayered constructs, a polymeric supportive frame also fulfills the tissue-specific role of inducing cell alignment by acting as a geometrical constraint. Choi and co-workers employed a composite tissue/organ building system for the recapitulation of skeletal muscle constructs by 3D bioprinting C2C12 myoblasts in a skeletal muscle dECM bioink and PCL.^41^ PCL was deposited at both ends of the 3D bioprinted cell-laden filaments to produce geometrical constraints which can further induce an alignment along the longitudinal direction. The printing environment was maintained at 18 °C to inhibit the gel transition of the dECM bioink during the biofabrication process. Muscle constructs were then incubated for 1 h at 37 °C to achieve full gelation after 3D bioprinting. The C2C12-laden dECM bioink was printed with different architectures and line widths. After 7 days of differentiation, MHC immunostaining showed the formation of mature multinucleated and aligned myotubes with characteristic striated band patterns. In addition, 3D bioprinted muscle constructs spontaneously generated visible contraction in response to electrical stimulation. Interestingly, the agrin preserved in the dECM induced the formation of acetylcholine receptor (AChR) clusters, as observed from the remarkable number of α-bungarotoxin (α-BTX) positive cells detected from the immunofluorescent analysis. In another study, Kim and co-workers employed integrated tissue-organ 3D bioprinting to fabricate skeletal muscle constructs by simultaneously printing a human muscle progenitor cell (hMPC) laden fibrinogen-based bioink, a sacrificial acellular gelatin hydrogel bioink, and a supporting PCL polymer.^145^ Cell-laden bioink was cross-linked with thrombin, while gelatin was dissolved during incubation at 37 °C to create microchannels through the construct (Figure 5Ai–iii)). After 7 days of culture, aligned MHC-positive myotubes were formed. Constructs were also evaluated *in vivo* via implantation in a rodent model of TA muscle defect. After 8 weeks, muscle constructs reached 82% of functional recovery and 85% of normal muscle force. Moreover, the bioprinted structures contributed to highly organized muscle tissue regeneration, while severe muscle atrophy and limited muscle regeneration were observed with nonprinted scaffold controls (Figure 5Av, vi). In addition, vascularization and host nerve integration were also observed, as confirmed by histological and immunohistological tests.

#### 4.3.2 Microfluidic-Assisted 3D Bioprinting

Microfluidic-assisted 3D bioprinting consists in using a microfluidic chip as an extruder for the deposition of hydrogel fibers. This strategy is often used in combination with coaxial nozzles, enabling the simultaneous delivery of both cross-linking solutions and a cell-laden hydrogel bioink through the outer and inner nozzle, respectively.^146−149^ As a result of such on-process cross-linking, a rigid and stable cell-laden hydrogel filament is formed, thus preventing fibers from spreading or collapsing during the deposition process. Hence, the fabrication of high-resolution 3D constructs can be successfully performed.^135,150^ Hydrogel-based bioinks employed in microfluidic-assisted 3D bioprinting require fast cross-linking properties to undergo immediate gelation at the tip of the coaxial extruder.^150−152^ To meet such biofabrication criteria, alginate-based bioinks were widely used for their instantaneous physical gelation properties.^42,153,154^ In one study, photocurable monoacrylated PEG-fibrinogen/alginate hydrogel was coaxially extruded with CaCl_2_ to 3D bioprint C2C12-laden constructs.^7^ High-resolution hydrogel filaments were successfully bioprinted in a 0–180° geometry and UV photo-cross-linked after fabrication (Figure 5Bi, ii). After 21 days of culture, striated myofibers were formed, and myotube contraction was visible without the application of external stimuli (Figure 5Biii, iv). Moreover, after 28 days of *in vivo* subcutaneous implantation in the back of immunocompromised mice, the constructs generated organized and mature muscle-like tissue.

#### 4.3.3 Direct 3D Bioprinting

Self-standing strands without supporting structure or on-processing cross-linking can be obtained by increasing the viscosity of the hydrogel bioinks. To this aim, a pristine hydrogel can be blended with other hydrogels to create a composite bioink. In one study, pristine GelMA was combined with other materials (i.e., alginate, cellulose, and PEGDA) to 3D bioprint C2C12 myoblasts.^155^ On day 11 of culture, pristine GelMA scaffolds appeared nearly flat, while composite hydrogels preserved their 3D structure. Indeed, in the phase of fabrication, GelMA-filaments collapsed and resulted in wider fiber diameter. Moreover, myoblast metabolic activities contributed to the degradation of pristine GelMA. As a result, proliferation, cell alignment, and myotube formation were enhanced in the composite hydrogels. Besides confining cells in a self-standing 3D bioprinted filament, muscle-cell alignment can be improved by specific bioink topographical cues obtained by extruding bioink through the nozzle. Indeed, hydrogel bioinks tend to undergo molecular orientation due to the intrinsic strain applied as the hydrogel is extruded through the nozzle.^40,156^ To control the fibrillar orientation degree of polymer molecules, two main 3D printing parameters are generally manipulated: pneumatic pressure (i.e., flow rate) and nozzle speed rate. In particular, higher speed rates may increase the stretching of extruded bioinks, thus leading to enhancement in the polymer chain orientation. The same result can be generated by applying a high flow rate. However, the bionks may flow in a random direction at the nozzle tip when a large amount of hydrogels solution is delivered at high-volume flow rates. Therefore, such effect might reduce the overall fiber anisotropy. In this respect, Kim et al. modulated the fibril orientation of collagen-based bioink by properly tuning nozzle speed rate and flow rate (Figure 5Ci, ii).^157^ To further enhance the topographical cues, postprinting collagen fibrillation was performed by immersing the construct in a glycine/potassium chloride (Gly/KCl) buffer solution. 3D bioprinted scaffolds without fibrillation treatment were referred to as a control. After 14 days of culture, embedded C2C12 myoblasts were fully aligned toward the printing direction, while control scaffolds showed random orientation (Figure 5Ciii). In addition to inducing alignment into the polymeric fibrils, the wall shear stress induced during the 3D bioprinting process may also directly act on encapsulated cells by fluidically stimulating cell alignment. In one study, 3D bioprinting of GelMA hydrogel mixed with precultured C2C12 myoblasts was performed.^129^ The rationale behind this method consisted of using a preculturing period to develop an anisotropic cytoskeleton, which is likely more sensitive to external shear stress compared to spherically shaped cells. Indeed, the shear stress induced on uniaxially orientated cells can affect the extension of myoblast filopodia and eventually activate a specific signaling pathway. In this study, different preculturing periods (i.e., 3, 5, 7 days) were evaluated. Nonprecultured cell-laden GelMA bioink was kept as a control. On day 14, embedded C2C12 myoblasts precultured for 5 days showed higher alignment, as well as greater MHC and actin expression compared to controls.

#### 4.3.4 Hybrid 3D Bioprinting

Similar to hybrid electrospinning, hybrid hydrogel 3D bioprinting allows the fabrication of hierarchical constructs by deposition of cell-laden bioinks on aligned fibrous polymeric surfaces, which can be obtained by electro-assisted spinning or melt-plotting 3D printing techniques. As previously mentioned (see section 4.3), hydrogel bioinks play the role of cell-carrier by homogeneously releasing encapsulated muscle cells on the neighboring aligned polymeric fiber networks over the culture period.^158^ To this aim, bioink composition and properties are specifically tailored to induce hydrogel degradation when scaffolds are immersed in cell culture medium. Such approach guarantees an even cell distribution over the 3D scaffold environment, thus being an advantage over standard cell seeding procedures. To induce the anisotropic topography, the polymeric thermoplastic structure can be either 3D printed or electrospun following an aligned arrangement. Alternatively, fibers can be randomly deposited and then subsequently subjected to uniaxial stretching.^158,159^ A hybrid 3D bioprinting approach was employed by Yeo et al., who fabricated hierarchical structures by electrospinning aligned PCL fibers onto melt-plotted PCL macro-sized struts, followed by C2C12-laden alginate/PEO 3D bioprinting.^159^ After incubation, alginate and PEO dissolved, allowing homogeneous cell release on the fibrous PCL struts. After 7 days of culture, myoblasts seeded on aligned scaffolds displayed a bipolar stretching shape and mature sarcomeric structure. Moreover, to further mimic the architecture of the skeletal muscle bundle, cell-laden hierarchical structures were rolled in a cylindrical shape (Figure 5D). After 1 week, magnified SEM images revealed robust cell proliferation and alignment along the longitudinal fiber direction.

### 4.4 Hydrogel Extrusion

The hydrogel extrusion technique is an easy and cost-effective method to produce meter-long microfibers. Hydrogel solutions are continuously delivered by means of syringe pumps toward low-cost extruders such as syringe needles, synthetic tubing, or micronozzle arrays.^160^ Hydrogel microfibers can be obtained either by extrusion of precross-linked solution or by ejection into a cross-linking bath.^160,161^ In SMTE, such an approach has been used to produce both cell-free and cell-laden hydrogel microfibers.

#### 4.4.1 Cell-Seeded Extrusion

**Figure 6:**
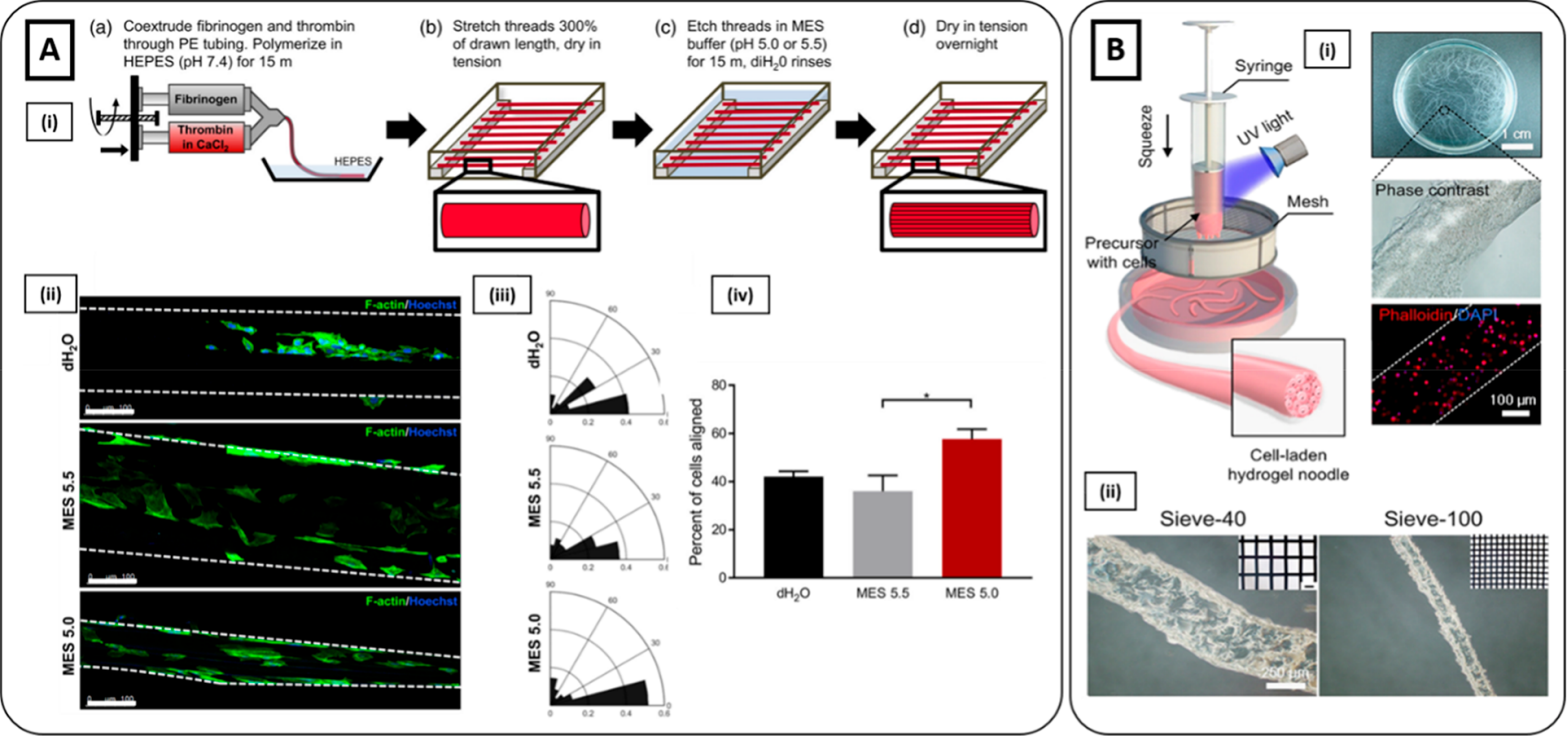
Hydrogel extrusion methods. (**A**i) Schematics of the fabrication of fibrin microthreads with MES etching (pH = 5.5 or 5.0). (ii) Immunostaining of phalloidin (green) and Hoechst (blue) showed higher myoblast elongation on fibrin microthreads treated with MES 5.0 compared to those treated with MES 5.5 and dH_2_O microthreads (control). Scale bar 100 μm. (iii) Nuclear orientation and (iv) the percent of total cells aligned along the microthreads direction (0°–15°) demonstrated higher preferential orientation along the long axis for MES 5.0 treated microthreads (Reproduced with permission from ref (165). Copyright 2020 WILEY-VCH Verlag GmbH & Co. KGaA, Weinheim). (**B**i) Schematics of the fabrication of hydrogel fibers inspired by the production process of traditional Chinese noodles. Phase-contrast and fluorescent images (phalloidin red, nuclei blue) showed cells successfully encapsulated into hydrogel microfibers. (ii) Brightfield microscopy images of microfibers obtained using a sieve with different pore sizes (300 μm left, 100 μm right) (Reproduced with permission from ref (169). Copyright 2019 The Authors, Springer Nature CCBY-NC-ND 4.0).

The hydrogel extrusion method for muscle cell seeding was first employed by Pins and colleagues, who fabricated fibrin microthreads by extruding in-line fibrinogen and thrombin/CaCl_2_ into a HEPES bath by means of polyethylene tubing.^161−163^ To enhance the topographical alignment, fibrin microthreads were subjected to static uniaxial stretching up to 200% of their initial length through a custom-made stretching device.^164^ Such postprocessing treatment promoted fibrin fibrils alignment, which in turn increased the overall mechanical properties and the cell orientation along the fiber axis up to 30% compared to not-stretched fibers. Besides uniaxial stretching, the potential of fibrin microthreads in promoting skeletal muscle cell alignment can be further enhanced by surface chemical treatments. Carnes et al. developed an etching method to induce the formation of aligned microgrooves on the surface of microthreads, by using a 2-(*N*-morpholino) ethanesulfonic acid (MES) acidic buffer at different pH values (i.e., pH = 5.5 and 5) (Figure 6Ai).^165^ Microthreads treated with deionized water were kept as a control. Surface characterization performed with atomic force microscopy (AFM) and SEM indicated higher microgrooved alignment for microthreads treated with MES at pH = 5. Enhancement in topographical cues was also reflected at the cellular level with higher C2C12 myoblasts nuclear orientation and alignment than the other conditions. (Figure 6Aii–iv).

#### 4.4.2 Cell-Laden Extrusion

Postprocessing treatments used to cross-link extruded fibers are generally not suitable for maintaining cell viability. Hence, extrusion techniques aiming to encapsulate cells need to employ methods to favor an efficient microfibers cross-linking and simultaneously ensure cell-compatible conditions.^160,166^ To generate myoblasts-laden hydrogel microfibers, Li et al. introduced an alternative extrusion method inspired by the approach used to produce Chinese noodles.^167^ C2C12 myoblasts were suspended in a solution of GelMA-PEGMA, loaded into a syringe, and exposed to UV light for photo-cross-linking. Then, the cross-linked hydrogel solution was squeezed through a sieve to fabricate C2C12-laden microfibers (Figure 6Bi). The diameter of cell-laden microfibers was tuned by using sieves of different pore sizes. For instance, sieves with 100 and 300 μm pore sizes were used to fabricate microfibers with a diameter of 322 and 129 μm, respectively (Figure 6Bii). A high number of alive cells (>90%) was detected for both pore-size sieves, demonstrating that the squeezing process preserved cell viability. Despite extrusion methods having been classified as simple and facile techniques, complex cell-laden fiber structures can be produced by coupling the spinning system with specific components. For example, the extruder can be connected with a kinetics static mixer (KSM), which is a sequential array of helicoidal mixing elements that induce a spatial-periodic deformation of two or more hydrogel solutions to generate an internal layered microstructure. In one study, the extrusion method was combined with a KSM in order to fabricate muscle-like hierarchical structures by simultaneously delivering alginate and GelMA/alginate/C2C12 solutions.^168^ Specifically, multilayer fibers were obtained by intercalating a myoblast-laden layer with physical barriers composed of pristine alginate. Cells exhibited high viability both postextrusion and after 28 days after culture. C2C12 myoblasts elongated within GelMA/alginate fibers, physically constrained by alginate barriers that prevent cells from migrating to neighboring layers. After 28 days of culture, C2C12 myoblasts differentiated into myotubes expressing MHC and sarcomeric actin.

### 4.5 Hydrogel Microfluidic Spinning

Microfluidics involves a wide range of advanced technologies which enable the precise manipulation of fluids at the microscale within a broad spectrum of designed channel-based configurations.^170^ Microfluidic chips with high resolution and complex designs can be easily produced by low-cost methods, including soft lithography techniques (e.g., PDMS and SU-8 molds processing) and nonsoft lithography techniques (e.g., xurography, micromilling, laser jet).^171^ Over the last decades, microfluidic technology has shown significant results in biomedicine and in the diagnostic field.^172−174^ For instance, organ-on-chips (OOCs) have been employed as a platform to simulate the physiological and pathological tissue microenvironment or to perform high-throughput drug screening or toxicology.^174−177^ Besides, microfluidics also emerged as a fascinating approach for the microengineering of hydrogels, including continuous fabrication of hydrogel microfibers.^178−182^ Hydrogel solutions are injected into the inlet of a microfluidic chip, delivered through microchannels in a laminar flow, and directly extruded from the outlet of microchannels or embedded syringe needles or glass capillaries.^170^ Once extruded, hydrogel solutions can be rapidly cross-linked by various gelation methods, including UV light, ionic or chemical cross-linking, and solvent exchange, thus enabling the fabrication of meter-long hydrogel microfibers in a relatively short time.^24^ Individual fibers can also be assembled in 3D fibrous structures by reeling or weaving techniques.^25,170^ Based on such promising features, microfluidic spinning is considered an attractive tool for the fabrication of fibrous tissues and organs, such as skeletal muscle tissue constructs.^183^ In this frame, microfluidic spinning has been employed to produce hydrogel microfibers for (i) muscle cell-seeded alignment on the anisotropic surface and (ii) for muscle-cell encapsulation.

#### 4.5.1 Cell-Seeded Microfluidic Spinning

**Figure 7:**
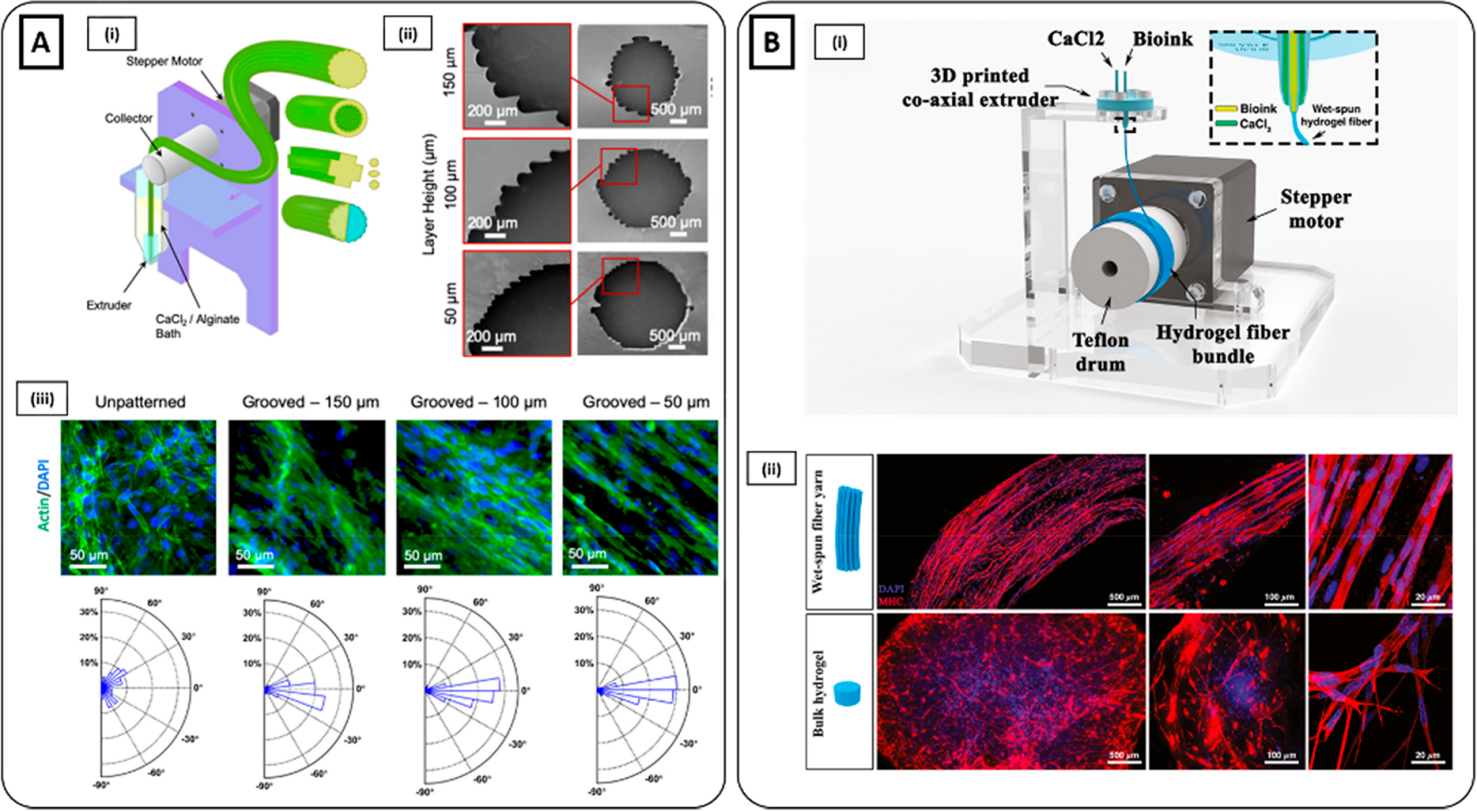
Hydrogel microfluidic methods. (**A**i) Schematic illustration of the wet-spinning device and the fabrication process of solid and hollow grooved hydrogel fibers with various cross-sectional shapes. (ii) SEM images of the microfluidic extruder with different grooved dimensions. (iii) Quantitative analysis of alignment for C2C12 myoblasts on the grooved fibers with three different groove sizes and unpatterned fibers based on the confocal microscopy images (F-actin green, nuclei blue). Cells on unpatterned fibers showed a random distribution. In contrast, myoblasts demonstrated alignment toward the grooves, which increased by decreasing the groove size from 150 to 50 μm (Reproduced with permission from ref (188). Copyright 2020 American Chemical Society). (**B**i) Schematic representation of microfluidic spinning setup for the fabrication of hPM-laden hydrogel yarns. (ii) MHC (red) and nuclei (blue) staining of hPM-laden yarn and bulk sample (control) after 15 days of culture. Cell-laden yarns exhibited parallelly aligned MHC positive myotubes, while bulk samples showed a similar MHC expression but with a random myotube arrangement (Reproduced with permission from ref (193). Copyright 2020 The Authors, John Wiley and Sons CCBY-NC-ND 4.0).

Hydrogel microfibers with different morphologies can be fabricated by manipulating the design of the microfluidic outlet. Hydrogel extruded from the shaped outlet undergoes immediate cross-linking, thus allowing microfibers to maintain the molded morphology. Such microfibers can assume different morphologies to fulfill the biomimetic features required to address the specific tissue engineering application.^181^ To reproduce a muscle tissue construct, microgrooved microfibers are employed to guarantee proper alignment and maturation for skeletal muscle cells seeded on the surface. Micropatterned fibers are generally produced by extruding a hydrogel solution through grooved microfluidic channels.^184,185^ In one study, microgrooved GelMA microfibers were generated using a microfluidic device consisting of a grooved cylindrical channel and immediately cross-linked by directly flowing into a cold ethanol bath (21 °C).^186^ Smooth GelMA microfibers were kept as a control. C2C12 myoblasts elongated along the groove direction after 3 days of culture, while they spread randomly on controls. Ebrahimi et al. further investigated the effect of GelMA microgrooved fibers on myoblast cells by adding recombinant rat agrin as a supplement for differentiation medium.^187^ The combination of topographical cues with agrin treatment was found to upregulate AChR and dystrophin expression in differentiated myotubes. Moreover, myotube maturation and functionality were enhanced by improved contractility under electrical stimulation. By adjusting the microfluidic design, micropatterned fibers can assume different cross-sectional shapes, such as helicoidal, circular, or flat. Furthermore, groove dimensions can be modulated to investigate the effect of different micropattern sizes on myoblast alignment and maturation.^184^ Mirani et al. 3D-printed a PLLA-based microfluidic chip with different groove dimensions to obtain alginate-based fibers with microgrooves in the range of 50–150 μm (Figure 7Ai, ii).^188^ It was observed that smaller grooves (50 μm) promoted C2C12 myoblast cell alignment and myogenic differentiation compared to wider structures (150 μm) (Figure 7Aiii). Moreover, such fabrication methods allowed the successful encapsulation of conductive material particles, which may further induce muscle tissue differentiation by improving cell-to-cell electrical transmission.

#### 4.5.2 Cell-Laden Microfluidic Spinning

Due to the limited time necessary for solidification, fast gelation methods are required when using microfluidic devices.^189^ In this frame, alginate has been widely employed for microfluidic spinning applications thanks to its instantaneous ionic cross-linking properties.^24,190,191^ Alginate-based microfibers for SMTE can be produced by coaxially delivering hydrogel/myoblast solution and cross-linking agent (e.g., CaCl_2_).^192^ For instance, Costantini et al. developed a muscle structure by coaxially spinning human primary myoblasts (hPMs) suspended in a monoacrylate-PEG fibrinogen/alginate and CaCl_2_ solution.^193^ Cell-laden microfibers were collected on a rotating round-shaped drum to obtain fibrous yarns mimicking the anisotropic muscle organization (Figure 7Bi). Cell-laden bulk hydrogels obtained with the same hydrogel composition were kept as a control. After 2 weeks, cell-laden yarns exhibited parallel aligned MHC positive myotubes, while bulk samples showed a similar MHC expression but a random myotube arrangement (Figure 7Bii). Furthermore, the performance of cell-laden yarns was also tested *in vivo* by implantation in a TA defect in an immunocompromised mouse model. After 20 days, the constructs revealed high regeneration capacity, formation of new blood vessels, and neuromuscular junctions (NMJs). Although its fast gelation properties are suitable for microfluidic fabrication techniques, alginate lacks adhesive motifs to ensure cell adhesion.^146^ Even though ECM-mimicking hydrogels can guarantee a more cell-friendly environment, they are associated with several shortcomings, mainly related to long gelation time and low mechanical properties, which in turn may interfere with the fabrication of hydrogel microfibers toward the microfluidic spinning approach.^16^ Therefore, the generation of core–shell microfibers encapsulating ECM-like hydrogels in thin and tubular alginate-based shells offers a promising platform able to couple a cell-compatible microenvironment with appropriate mechanical strength and stability within fiber-shape structures.^194^ In this frame, Onoe et al. developed a microfluidic device generating a double-coaxial laminar flow stream consisting of a core stream of a wide variety of ECM-like hydrogel solutions (e.g., collagen and fibrinogen) and a shell stream of alginate, solidified upon contacting with CaCl_2_.^26^ Such a microfluidic approach allowed successful encapsulation of the core within the sheath, thus preventing diffusion of cells and ECM-like hydrogels during the fabrication process. After cross-linking the core by thermal gelation at 37 °C, the shell was selectively digested by alginate lyase to obtain microfibers entirely made of collagen. Different cell sources (e.g., human umbilical vein endothelial cells (HUVECs), fibroblasts (NIH/3T3), nervous cells) were successfully encapsulated, exhibiting tissue-specific marker expressions. To fabricate skeletal muscle tissue constructs, C2C12 myoblasts were also encapsulated into microfibers.^195^ After 4 days of culture, myoblast-laden microfibers showed an increased the ratio of mature myotube-like cells compared to the 2D control group. To avoid the use of alginate, photo-cross-linkable hydrogels were also employed to spin muscle-laden microfibers. Wang et al. fabricated hollow microfibers using a microfluidic coaxial needle to simultaneously extrude a solution of GelMA/gelatin-C2C12, together with PVA used as a sacrificial material.^196^ Microfibers were directly dispensed in an ice-cold PBS bath at 4 °C and to induce thermal gelation. Then, C2C12-laden microfibers were further stabilized by photo-cross-linking using UV light irradiation. C2C12 myoblasts were successfully encapsulated and revealed high viability for up to 7 days.

## 5 Mechanical and Electrical Stimulation of Hydrogel-Based Fiber Scaffolds for SMTE

**Figure 8:**
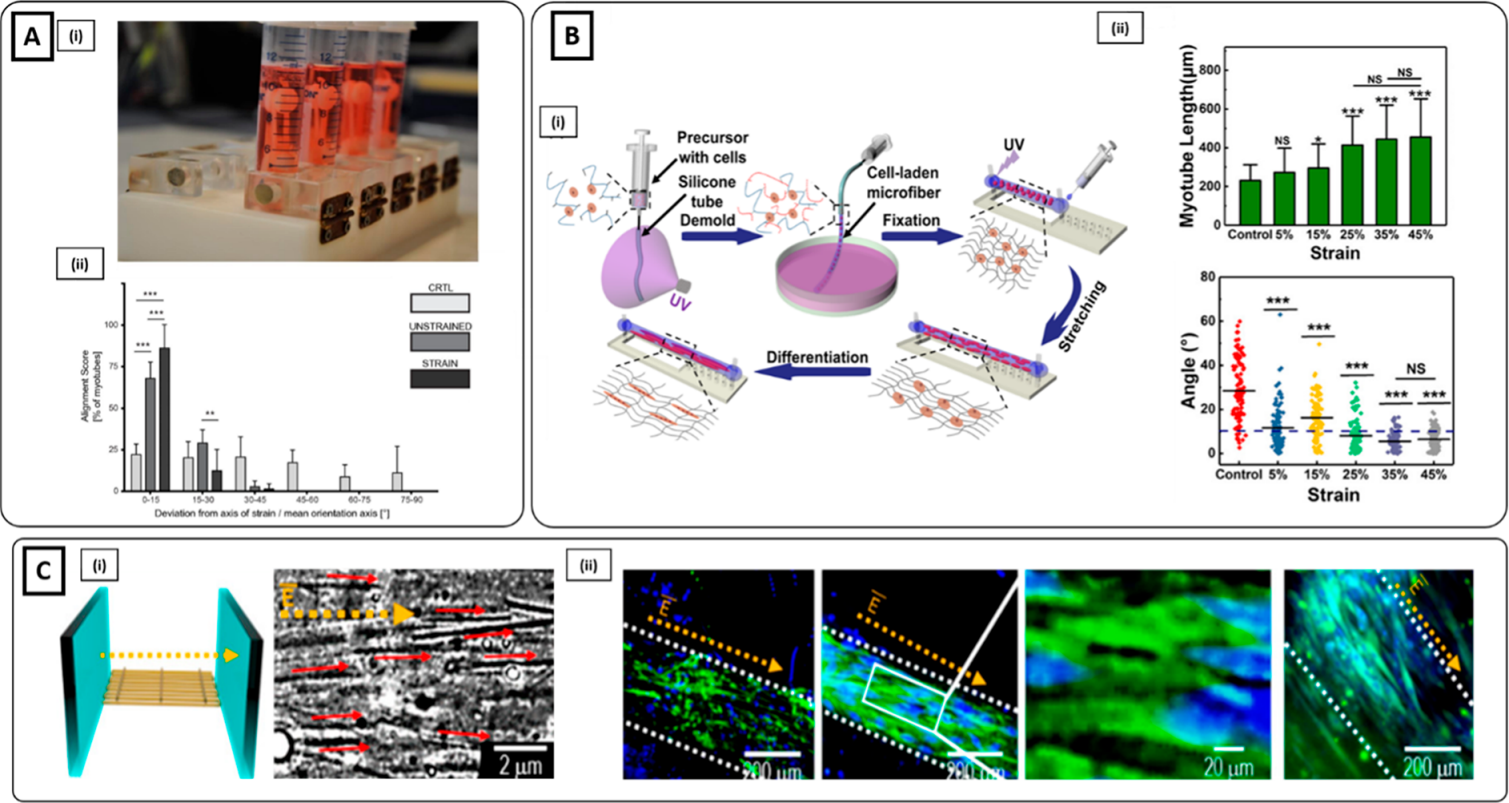
Mechanical and electrical stimulation on hydrogel-based fibers for SMTE. (**A**i) C2C12-laden ring-shaped fibrin scaffold obtained by micromolding subjected to uniaxial mechanical stimulation through a spool–hook system working via magnetic force transmission. (iii) Assessment of myoblast alignment along the uniaxial direction revealed higher cell alignment for strained samples compared to unstrained (Reproduced with permission from ref (5). Copyright 2015 Elsevier). (**B**i) Schematic representation of the cell-laden microfiber fabrication process and the uniaxial stretching of C2C12 myoblast laden microfibers. (ii) Cell-laden microfibers displayed an enhancement of myotube length and cell orientation when subjected to a strain higher than 35%. Scale bar 200 μm (Reproduced with permission from ref (72). Copyright 2019 American Chemical Society). (**C**i) Schematics describing the direction of the electric field and the C2C12-laden GNWs/collagen scaffold and optical images showing the parallel distribution of GNWs. (ii) Immunostaining of MHC (green) and DAPI (blue) revealed alignment of myoblast along the electrical field direction (Reproduced with permission from ref (197). Copyright 2019 American Chemical Society).

The alignment of muscle cell precursors provided by fiber-shape structures can be coupled and enhanced by applying external stimuli to thoroughly recapitulate the skeletal muscle *in vivo* conditions. In this frame, mechanical stimulation plays a critical role in skeletal muscle development and maturation. During the embryogenesis and during postnatal physical activities (e.g., walking), skeletal muscle is subjected to mechanical stretching that allows for the alignment of myofibers and in turn leads to skeletal muscle tissue maturation. In particular, the mechanical forces trigger the mechanotransduction process, which in turn induces a rearrangement of the cytoskeleton in the direction of the mechanical stimulus.^76^ In SMTE, uniaxial strain resulted in positive myogenic outcomes such as increased myotube alignment, fusion, and differentiation. Mechanical stimulation has been used for hydrogel fiber-based skeletal muscle constructs by applying a tensile force and producing a stretching stimulus along the fiber direction. Therefore, different bioreactor systems can be used to grab the extremities of hydrogel fibers and produce uniaxial stretching under different modalities (i.e., static or dynamic) and in a wide range of amplitudes and frequencies to promote skeletal muscle formation.^112^ In one study, molded C2C12-laden fibrinogen ring-shaped structures were subjected to mechanical stretching (i.e., 10% static strain for 6 h followed by 18 h rest phase at 3% static strain) induced by a custom-made spool–hook system working via magnetic force transmission (Figure 8A).^5^ Compared to unstrained samples, mechanically stimulated constructs showed higher myotube alignment and maturation in terms of sarcomeric pattern formation and myotube diameter and length. Such an improvement was also reflected at the molecular level as upregulation of myogenic genes (e.g., myoblast determination protein 1 (MyoD) and myogenin) was obtained. Similarly, Chen et al. stimulated C2C12/GelMA 10 cm long molded microfibers under uniaxially stretching at different strain ratios (i.e., 5, 15, 25, 35, 45%) by using a pillar-based device (Figure 8Bi).^72^ A 0% strain ratio was kept as a control group. As a result, up to 35% of strain ratios promoted C2C12 myoblasts orientation and myotubes maturation (Figure 8Bii). In another study, C2C12-seeded electrospun fibrin microfibers were cultured for 7 days in custom-built bioreactor units to investigate the impact of a static strain (i.e., 10% strain for 6 h per day) and a cyclic strain (i.e., 10% strain at the frequency of 0.5 Hz for 6 h per day).^112^ Both static and cyclic uniaxial strains resulted in similar morphological and gene expression outcomes. Interestingly, a significant increase in myotube diameter, MHC coverage, and expression of key myogenic genes was observed in strained samples compared to the control when the mechanical stimulation was applied at days 5–7 rather than days 3–7 of culture. Those findings highlighted that a significant myogenic differentiation could be achieved by applying uniaxial stretching at an advanced myoblasts maturation stage.

Besides mechanical stimulation, electrical stimuli have also been proved to be a fundamental foster myogenic differentiation.^77^ Indeed, it has been demonstrated that during the first stages of neonatal muscular development, the presence of an intact nerve is crucial for the proper growth and maturation of new myofibers. Moreover, mature musculature in *in vivo* is innervated by nervous structures, which provide further stimuli critical for the differentiation of satellite cells and the conversion of MHC isoforms.^76^ To enhance the pro-myogenic effects, electrical stimulation is generally coupled with electroconductive fillers, which are embedded into the hydrogel matrices. Moreover, similarly to mechanical conditioning, also electrical signals can favorably exploit the unidirectional morphology provided by microfibers structure. In this frame, Kim et al. used the 3D bioprinting technique to anisotropically orient gold nanowires (GNWs) in a C2C12-laden collagen-based bioink.^197^ After 14 days of culture, an external electric field was applied to the 3D bioprinted scaffolds to induce an electrical stimulus along the fiber direction (Figure 8Ci). Compared to the nonstimulated samples, electric field signals induced higher myoblast alignment and myotube formation. Such an effect was obtained by the simultaneous action of the myogenic inducing power of external electric stimulation and by the enhancement of GNWs orientation due to the induced dipole moment and the geometrical shape (Figure 8Cii). Similarly, Wang et al. 3D bioprinted C2C12 myoblasts in a GelMA-based bioink combined with electroconductive PEDOT nanoparticles.^198^ The scaffolds were further subjected to electrical stimulation (5 V amplitude, 1 Hz frequency) over 4 h per day. The electrical stimulation promoted cell rearrangement and formation of myotubes after 10 days of culture. Furthermore, myogenic differentiation was also observed as demonstrated from the expression of myogenin and desmin.

## 6 Advanced Hydrogel-Based Fiber Models for Skeletal Muscle Tissue Interfaces

**Figure 9:**
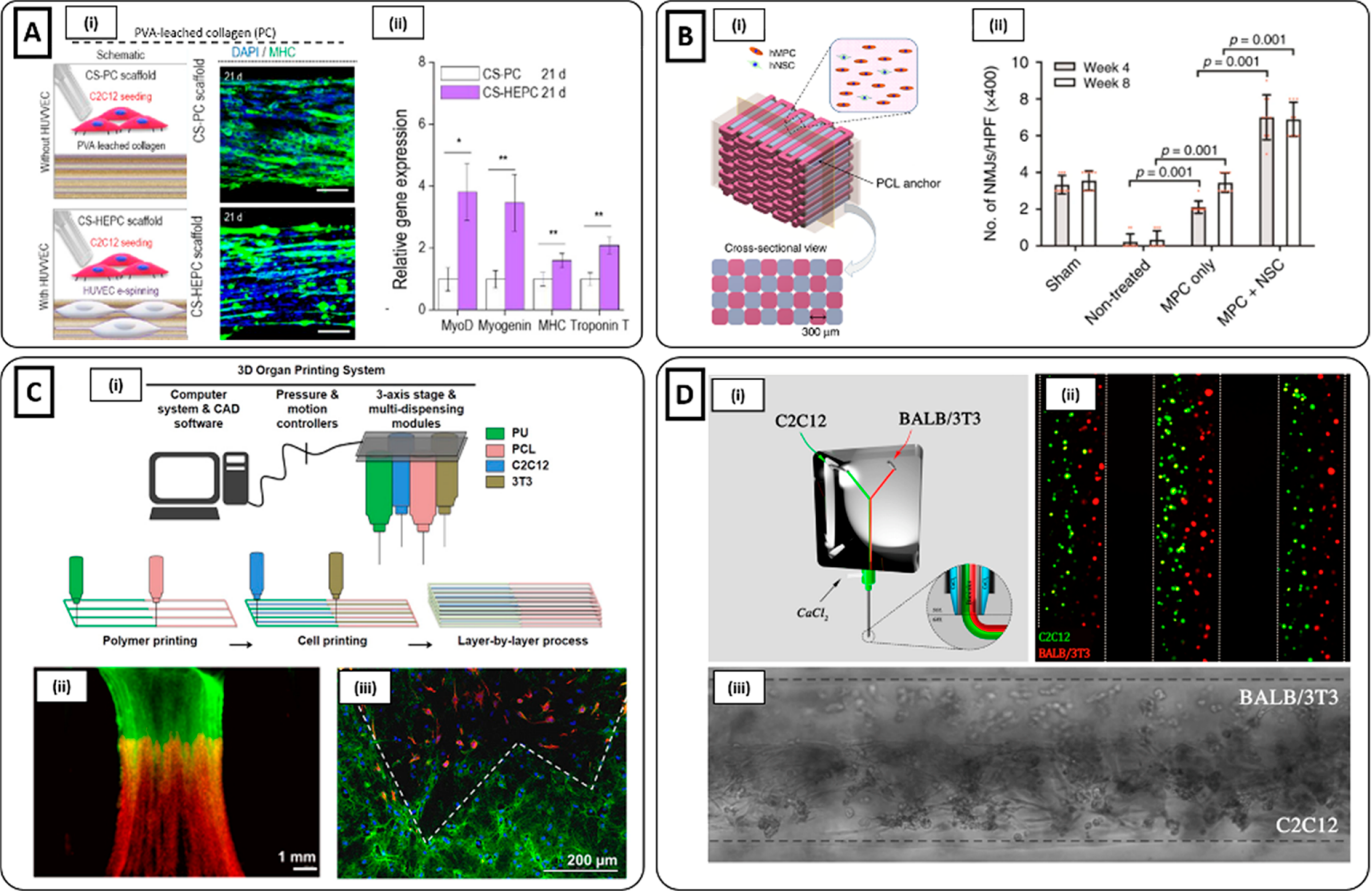
Advanced hydrogel fiber-based methods for the fabrication of skeletal muscle tissue interfaces. (**A**i) Schematic of C2C12-seeded alginate/PEO electrospun scaffold (CS-PC), C2C12-seeded on HUVEC-electrospun alginate/PEO scaffold (CS-HEPC), and corresponding fluorescent images of MHC (green) and nuclei (blue) on day 21. Enhanced MHC expression and (ii) relative myogenic gene expression was observed for C2C12/HUVEC scaffolds compared to those with C2C12-only (Reproduced with permission from ref (205). Copyright 2020 Elsevier). (**B**i) Schematic of hMPC/hNSC-laden 3D bioprinted scaffold for the fabrication of neuronal/muscle interface. (ii) A higher number of NMJs was detected on hMPC/hNSC-laden 3D bioprinted scaffold (MPC+NSC) compared to hMPC-laden 3D bioprinted scaffold (MPC) after 8 weeks of implantation (Reproduced with permission from ref (144). Copyright 2020 The Authors, Springer Nature CCBY-NC-ND 4.0). (**C**i) Schematic of the 3D integrated organ printing (IOP) system for the fabrication of MTU. (ii) Fluorescently labeled 3D bioprinted MTU constructs (green C2C12 myoblasts; red NIH 3T3 fibroblasts; yellow interface region). (iii) C2C12 myoblasts and NIH 3T3 fibroblasts expressed desmin (red) and collagen type I (green), respectively. At the interface region, cells created a pattern reliable with a native tissue interface (Reproduced with permission from ref (143). Copyright 2015 IOP Publishing). (**D**i) Multicellular 3D bioprinting through a Y-shaped microfluidic printing head. (ii) C2C12 myoblasts (green) and BALB/3T3 fibroblasts (red) were simultaneously extruded to obtain a Janus-like configuration which (iii) retained high compartmentalization after 5 days of culture (Reproduced with permission from ref (7). Copyright 2017 Elsevier CCBY-NC-ND 4.0).

To properly exert its contractile function, skeletal muscle tissue is connected to the neural, vascular, and connective compartments, respectively. Such tissue interaction originates from complex interfaces that present a high degree of structural organization. They are orchestrated by multiple signaling pathways that enable a simultaneous physiological collaboration to successfully pursue skeletal muscle function.^199^ For instance, the interface between muscle and nervous tissues is represented by highly specialized NMJs, which ensure signal transfer across the excitatory synapse between a motor neuron and the skeletal myofiber, thus allowing effective muscle contraction.^200^ Skeletal muscle is also irrorated by a dense capillary network, which provides transport of oxygen and nutrients along with the secretion of a wide spectrum of angiocrine factors in order to regulate tissue homeostasis.^200,201^ Moreover, muscle tissue is interpenetrated by connective tissue containing fibroblasts cells, which supplies an elastic framework to support synergistic contractile function and is also involved in the secretion of ECM components and growth factors to ensure myoblast differentiation.^202^ Finally, skeletal muscles are also connected to tendons to form the myotendinous junction (MTJ), which allows the transmission of force generated by the muscle through the tendon onto the bone to produce a movement.^203,204^ Considering the key role of such tissue interfaces, it is of paramount importance to reproduce their biological and architectural complexity *in vitro* to enhance skeletal muscle maturation and functionality. Moreover, the incorporation of nervous/vascular/connective tissue in *in vitro* may enable efficient and rapid integration of the surrounding tissue upon *in vivo* implantation, guaranteeing the long-term survival and functionality of the constructs. Thus, SMTE strategies must be addressed not only to recapitulate cell alignment and maturation but also to mimic the complex interfaces between muscle and neighboring compartments (Table 3). Yeo et al. developed a vascularized muscle tissue construct by culturing C2C12 myoblasts on HUVEC-laden alginate/PEO nanofibers produced by the cell-electrospinning process (Figure 9Ai).^205^ Compared to the C2C12-only scaffolds, HUVEC-C2C12 constructs enhanced myoblast differentiation with a higher degree of MHC and sarcomeric α-actin expression, along with improved myogenic-specific gene expressions (e.g., MyoD, troponin T, MHC, and myogenin) (Figure 9Ai, ii). Kim et al. engineered a neural integration into skeletal muscle by 3D bioprinting human muscle progenitor cells (hMPCs) and human neural stem cells (hNSCs) in a fibrinogen-based hydrogel.^144^ The cell-laden hydrogel was coextruded with PCL forming a supporting frame and a sacrificial gelatin-based ink to create microchannels (Figure 9Bi). After 3D bioprinting, constructs were cultured in growth medium overnight and then switched to differentiation medium supplemented with aprotinin. *In vivo* implantation in a TA muscle defect of a rodent model revealed that muscle-nerve constructs accelerated the restoration of skeletal muscle function compared to the only muscle constructs, as confirmed by the higher number of the MHC positive myofibers. Moreover, the hNSCs also differentiated into neurons and glial cells, contributing to inducing *in vivo* NMJ formation (Figure 9Bii). By employing multimaterial 3D bioprinting, the development of constructs with an improved spatial organization can be obtained by precise and controlled positioning of multiple hydrogels and different cell types.^133^ In this frame, Merceron et al. employed multimaterial 3D bioprinting to recreate a bicompartmental scaffold to reproduce the myotendinous unit (MTU).^143^ In particular, PU and PCL were printed to create a scaffolding structure, which was subsequently filled with C2C12-laden and NIH/3T3-laden fibrin-based bioink to generate the muscle and tendon side, respectively (Figure 9Ci, ii). Such an approach allowed the reproduction of the MTU mechanical heterogeneity by using thermoplastic polymers, which replicated the elastic and stiff nature of muscle and tendon, respectively. Moreover, the cellular complexity was also successfully reproduced. C2C12 myoblasts aligned along the fiber axis expressed both desmin and MHC and started to show multinucleation; while on the tendon side, initial deposition of collagen type I was observed (Figure 9Ciii). At the interface region, cells were able to create a self-organization pattern reliable with a native tissue interface. Moreover, gene expression profiles of the 3D bioprinted MTU constructs (versus muscle-only printed constructs) revealed an increase in the focal adhesion markers responsible for upregulating the MTJ region (e.g., vinculin, talin). An alternative approach to multimaterial 3D bioprinting, which relies on the use of multiple printing heads, microfluidic assisted 3D bioprinting can be employed hydrogels to recreate complex tissue architectures by simultaneous or alternative delivery of different cell-laden bionks.^151,152^ Costantini et al. developed a microfluidic device bearing a Y-junction (2 inlets, 1 outlet), which was fluidically connected with a coaxial extruder to deliver different types of bioinks in a precise and controlled manner by programming external microfluidic pumps (Figure 9Di).^7^ Such an approach was employed to reproduce connective tissue interface by fabricating Janus-fiber constructs obtained through the simultaneous delivery of C2C12 myoblasts and BALB/3T3 fibroblasts, both suspended in a photocurable PEG/alginate bioink (Figure 9Dii). As confirmed from fluorescence analysis, C2C12 and BALB/3T3 were finely compartmentalized within the hydrogel fibers and the Janus flow pattern was perfectly retained following gelation. Moreover, after 5 days of culture, C2C12 myoblasts started to form myotubes exclusively on the side of the hydrogel fibers in which they were compartmentalized (Figure 9Diii). The microfluidic chip design can also be manipulated to realize the one-step formation of complex architectures. To reproduce vessel-skeletal muscle architecture, Liu et al. developed a multiple-inlet microfluidic chip to deliver an alginate-based solution sandwiched by two streams of calcium chloride.^206^ The modulation of the flow rate ratio of the CaCl_2_ allowed to produce a double-folded hollow microfiber paralleling a straight microfiber. The straight and sine-wave channels served as a mimicking muscle bundle structure, surrounded by convoluted capillaries and anastomotic vessels. To validate the system, HSMCs and HUVECs were selected as cell sources, embedded into an alginate solution and spun. Cell viability assessment showed a high number of live cells (>90%) for both cell types.

**Table 3:** Hydrogel Fiber-Based Methods for the Biofabrication of Skeletal Muscle Tissue Interfaces

skeletal muscle interface	fiber-based biofabrication technique	cell types	*in vitro*/*in vivo* main outcomes	ref
vessel/muscle interface	cell/hybrid electrospinning	C2C12/HUVECs	enhancement of MHC and sarcomeric α-actin expression for HUVEC-C2C12 construct compared to those containing only muscle cells	(205)
vessel/muscle interface	microfluidic spinning	C2C12/HUVECs	fabrication of biomimetic structure formed by convoluted capillaries around a muscle bundle	(206)
			high viability (>90%) of both C2C12 and HUVECs	
neuromuscular junction (NMJ)	hybrid 3D bioprinting	hMPCs/hNSCs	integration of hNSCs improved skeletal muscle restoration upon *in vivo* implantation in TA rat defect	(144)
			differentiation of hNSCs into neurons and glial cells	
			*in vivo* innervation following NMJ formation	
myotendinous junction (MTJ)	hybrid 3D bioprinting	C2C12/NIH 3T3	recapitulation of MTJ mechanical and biological heterogeneous complexity	(143)
			myotubes formation and deposition of collagen type I at the muscle and tendon side, respectively	
			cell-organization pattern at the interface region	
			increase of focal adhesion markers responsible for upregulating MTJ compared to only muscle-side	
connective tissue/muscle interface	microfluidic-assisted 3D bioprinting	C2C12/BALB 3T3	fine compartmentalization of C2C12 myoblasts and BALB/3T3 fibroblasts in a Janus fiber configuration	(7)
			formation of myotubes exclusively in the compartmentalized region after 5 days of culture	

## 7 Conclusions and Future Perspectives

The main key factors of scaffold-based SMTE relied on the ability to mimic as closely as possible the native microenvironment of the skeletal muscle tissue. To this aim, engineered scaffolds should provide highly biocompatible and cell-supportive features along with proper anisotropic geometrical cues. Herein, hydrogel-based microfibers constitute an ideal platform to assist skeletal muscle tissue development. In this review, an overview of the biofabrication techniques to produce hydrogel microfibers for SMTE was presented, ranging from old-fashioned to new upcoming strategies. Moreover, in Table 4 the main advantages and disadvantages of such methods are also highlighted. A wide range of microfiber diameters can be obtained, from an ECM-protein size dimension (∼0.5 μm) to higher dimensions (∼500 μm), thus providing suitable topographical cues to favor myoblast alignment and differentiation. Microfibers can be produced by processing a wide variety of hydrogels from both natural and synthetic sources to recapitulate the biological features of the ECM microenvironment and provide excellent myogenic cues. Moreover, microfibers enable the recapitulation of the mechanical properties of skeletal muscle tissue by tailoring fabrication parameters (e.g., hydrogel concentration, cross-linking density, time of cross-linking), postprocessing treatments (e.g., uniaxial stretching), or by coupling supporting thermoplastic materials to form hierarchical structures or core–shell composite constructs. In addition, microfibers can be easily handled as building blocks to be further arranged and assembled to recapitulate a precise anisotropic alignment, thus developing fibrous 3D constructs with physiologically relevant features. An advanced recapitulation of the native-like microenvironment has been achieved by combining the engineered muscle construct with the connected tissue interfaces. To this aim, another approach is represented by the application of external stimuli to simulate the *in vivo* conditions.^207^ For example, to mimic the excitatory signal transmission, electrical stimuli can be applied. Alternatively, electroconductive materials, nanoparticles, or nanorods can be incorporated within the hydrogel matrix. Moreover, microfibers can also be subjected to uniaxial mechanical stimulation by means of specific bioreactors or stretching devices enabling the application of tensile stress with different ranges of amplitude and frequencies to recapitulate the *in vivo* muscle locomotion. Future developments in the field of hydrogel-based microfibers might be achieved by approaching 4D biofabrication methods. Such tissue engineering trend comprises various fabrication technologies that aim to achieve a desired structure or morphology by a programmable shape-transformation of preliminary fabricated 3D constructs.^208^ 4D biofabrication methods mainly rely on the use of stimuli-responsive or smart hydrogels.^209^ Such biomaterials can respond to external stimuli (e.g., pH, temperature, humidity, light, electricity, and magnetic fields) with swelling/deswelling processes, thus leading to morphological changes. Hence, smart hydrogels represent an intriguing choice for the fabrication of engineered muscle tissue microfibers.^169,210,211^ Indeed, in the case of fiber-shaped constructs, hydrogel swelling and shrinking mechanisms might occur preferentially along the longitudinal fiber axis, leading to anisotropic elongation and contraction.^212^ Moreover, such stimuli can be periodically applied to produce a cyclic deformation which can be subsequently translated into movement-like mechanisms.^212,213^ Besides the reproduction of mechanical stimuli, smart hydrogels can also be involved in the fabrication of constructs with higher tissue complexity and biomimetic features.^52,214^ For instance, Apsite et al. fabricated an alginate/PCL electrospun flat scaffolds that underwent thorough morphological deformation in an aqueous environment with different Ca^2+^ ion concentrations and self-rolled into a biomimetic muscle-like bundle to encapsulate myoblasts.^119^ Another step forward in this tissue engineering field consists of fabricating fibrous 3D scaffolds directly at the injury site.^69^ To date, *in situ* injectable hydrogels have been widely explored, showing high capacity in functionally regenerating skeletal muscles. On the other hand, in *situ* biofabrication approaches just made the first appearance in SMTE. *In situ* biofabrication may provide the exceptional advantage of precisely covering the geometry of a skeletal muscle defect caused by VML injuries, which are generally characterized by irregular shapes and sizes. Moreover, such approach would eliminate the need for additional surgeries necessary for the *in vivo* implantation of prefabricated constructs. Recently, Russel et al. developed a hand-held partially automated extrusion-based 3D bioprinter capable of delivering hydrogel strands directly into the VML injury site.^215^*In situ* 3D bioprinted scaffolds enabled the reconstruction of the defect site by supporting myogenesis and improving skeletal muscle hypertrophy upon injury. Such findings represent an innovative approach that can potentially lead to enormous advances in the treatment of traumatic injuries and in the field of SMTE.

**Table 4:** Advantages and Disadvantages of Hydrogel-Based Fiber Biofabrication Techniques for SMTE

hydrogel-based fiber biofabrication technique	advantages	disadvantages	ref
molding	cost-effective	time-consuming process	(74)
	facile	not suitable for the fabrication of complex structure	
	intuitive		
	reproducible		
electrospinning	production of nanofibers with the scale size of the ECM proteins	hydrogel selection limited by the spin viscosity range to fabricate defect-free fibers	(120, 122, 123)
	suitable to combine with fibrous thermoplastic scaffolds to enhance the mechanical properties and the anisotropic morphological cues	arduous compromise between spinnability and viability of encapsulated cells	
	fabrication of anisotropic structure by selecting specific collector configuration		
3D bioprinting	fabrication of highly defined physiologically relevant complex structures	time-consuming process	(7, 143)
	ability to use different hydrogels and cell types to mimic the heterogeneous skeletal muscle microenvironment	expensive and complex 3D bioprinting apparatus	
	suitable to combine with fibrous thermoplastic structures to enhance the anisotropic geometrical cues		
extrusion	cost-effective	hydrogel viscosity and postprocessing treatments hardly suitable for cell encapsulation	(165, 167)
	facile		
	continuous production of meter-long fibers		
	fiber diameters easily tunable by changing the syringe needles or sieve pore size		
	ability to create anisotropic microgrooved surfaces by chemical etching		
Microfluidic spinning	production of meter-long fibers in a relatively short time	not suitable to create complex architecture	(188, 193)
	production of microgrooved fiber by tailoring the design of the microfluidic chip outlet		
	ability to assemble fibers in anisotropic arrangement		

## Abbreviations

α-BTX: α-bungarotoxin
AFM: atomic force microscopy
AChR: acetylcholine receptor
bFGF-2: basic fibroblast growth factor-2
CNTs: carbon nanotubes
DC: direct current
dECM: decellularized ECM
DEP: dielectrophoresis
ECM: extracellular matrix
GelMA: gelatin methacryloyl
GNWs: gold nanowires
GO: graphene oxide
HLA: human leukocyte antigen
hMPC: human muscle progenitor cell
hPMs: human primary myoblast
HSMCs: human skeletal muscle cells
hSMPCs: human skeletal muscle progenitor cells
HUVECs: human umbilical vein endothelial cells
IGF-1: insulin-like growth factor-1
KSM: kinetics static mixer
MA: methacrylic anhydride
MES: 2-(*N*-morpholino) ethanesulfonic acid
MHC: myosin heavy chain
MTJ: myotendinous junction
MTU: myotendinous unit
MWNTs: multiwalled carbon nanotubes
MyoD: myoblast determination protein 1
NMJs: neuromuscular junctions
nSKM: neonatal skeletal myoblasts
OOCs: organ-on-chips
PAAm: polyacrylamide
PANi: polyaniline
PCL: polycaprolactone
PDMS: polydimethylsiloxane
PEDOT: poly(3,4-ethylene dioxythiophene)
PEG: poly(ethylene glycol)
PEGDA: poly(ethylene glycol) diacrylate
PEGS-M: PEG-*co*-poly(glycerol sebacate)
PEO: poly(ethylene oxide)
PIPAAm: poly(*N*-isopropylacrylamide)
PLA: polylactic acid
PLGA: poly(lactic-*co*-glycolic acid)
PLLA: polylactic acid
PMMA: poly(methyl methacrylate)
PU: polyurethane
PVA: poly(vinyl alcohol)
RGD: arginine–glycine–aspartic
rGO: reduced graphene oxide
SEM: scanning electron microscopy
SLA: stereolithography
SMTE: skeletal muscle tissue engineering
TA: tibial anterior
VEGF: vascular endothelial growth factor
VML: volumetric muscle loss
